# Enzymatic polymerization of enantiomeric _L_−3,4-dihydroxyphenylalanine into films with enhanced rigidity and stability

**DOI:** 10.1038/s41467-023-38845-3

**Published:** 2023-05-26

**Authors:** Yuhe Shen, Rongxin Su, Dongzhao Hao, Xiaojian Xu, Meital Reches, Jiwei Min, Heng Chang, Tao Yu, Qing Li, Xiaoyu Zhang, Yuefei Wang, Yuefei Wang, Wei Qi

**Affiliations:** 1 State Key Laboratory of Chemical Engineering, School of Chemical Engineering and Technology, Tianjin University, 300072 Tianjin, P. R. China; 2 Tianjin Key Laboratory of Membrane Science and Desalination Technology, 300072 Tianjin, P. R. China; 3 Collaborative Innovation Center of Chemical Science and Engineering (Tianjin), 300072 Tianjin, P. R. China; 4 Institute of Chemistry, the Hebrew University, Jerusalem, 91904 Israel; 5 State Key Laboratory of Component-based Chinese Medicine, Tianjin University of Traditional Chinese Medicine, 301617 Tianjin, China; 6 Haihe Laboratory of Modern Chinese Medicine, 301617 Tianjin, China; 7 Key Laboratory of Polymeric Materials Design and Synthesis for Biomedical Function, Soochow University, 215123 Suzhou, China

**Keywords:** Bioinspired materials, Organic molecules in materials science, Molecular self-assembly, Surface assembly

## Abstract

_L_−3,4-dihydroxyphenylalanine is an important molecule in the adhesion of mussels, and as an oxidative precursor of natural melanin, it plays an important role in living system. Here, we investigate the effect of the molecular chirality of 3,4-dihydroxyphenylalanine on the properties of the self-assembled films by tyrosinase-induced oxidative polymerization. The kinetics and morphology of pure enantiomers are completely altered upon their co-assembly, allowing the fabrication of layer-to-layer stacked nanostructures and films with improved structural and thermal stability. The different molecular arrangements and self-assembly mechanisms of the _L+D_-racemic mixtures, whose oxidation products have increased binding energy, resulting in stronger intermolecular forces, which significantly increases the elastic modulus. This study provides a simple pathway for the fabrication of biomimetic polymeric materials with enhanced physicochemical properties by controlling the chirality of monomers.

## Abstract (web summary)

_L_-3,4-Dihydroxyphenylalanine plays an important role in living system. Here, the authors investigated the effect of the molecular chirality of _L_-3,4-dihydroxyphenylalanine on the improved structural and thermal stability of self-assembled films by tyrosinase-induced oxidative polymerization.

## Introduction

Chirality, as one of the basic attributes of nature, is ubiquitous from the microscopic to macroscopic level^1,2^. Since the importance of stereochemistry in biochemical environments was proposed, chirality has become a hot issue and attracted the attention of the fields of chemistry, biology, and materials^3,4^. The study of chiral phenomena is not only of great significance to research fields such as catalysis, synthesis and separation, but also has important scientific and practical value to other scientific fields such as life science, pharmaceutical science, and material science^5–7^. Over the past decade, much effort has been devoted to investigating the chiral self-assembly of various molecules such as amino acids, peptides, and polymers into supramolecular materials with different structures and functions^8–10^. However, the effect of monomer chirality on the simultaneous covalent and noncovalent polymerization process has not been fully explored.

Recently, chirality at the surface and interface has attracted more and more attention^11–14^. The marine mussels are unique owing to their excellent ability to attach to a variety of wet surfaces and many studies have shown that their strong adhesion comes from the anchoring proteins secreted by the basement membrane, most of which contain a large amount of _L_−3,4-dihydroxyphenylalanine (_L_-DOPA)^15–18^. The DOPA-mediated bidentate hydrogen bonding, coordinative bonding with metals/ metal oxides, or covalent cross-linking are thought to be responsible for their wet stickiness^19–21^. DOPA or similar molecular units can firmly attach to almost all types of materials (including metals/alloys, glasses, inorganic oxides, and plastics), promising the preparation of high-performance adhesives with broad substrate compatibility^22–24^. At the same time, as the oxidation product of tyrosine, melanin formed by DOPA catalyzed by tyrosinase also plays an important role in life activities^25,26^. Over the past decade, a series of DOPA-containing synthetic polymers have been successfully developed for wet bonding and surface coating, such as polydopamine, and have attracted a wide range of applications in industrial, household and biomedical fields^27–30^. DOPA, as an intermediate oxidation product of a class of natural amino acids, inherits the chiral properties from tyrosine. However, the effect of chirality on its adhesion has not been reported. Studying the adhesion properties of chiral DOPA on the interface can better explain the adhesion mechanism of mussels on different surfaces, and provide theoretical guidance for the development and design of a new generation of robust adhesives and coating materials.

**Fig. 1:**
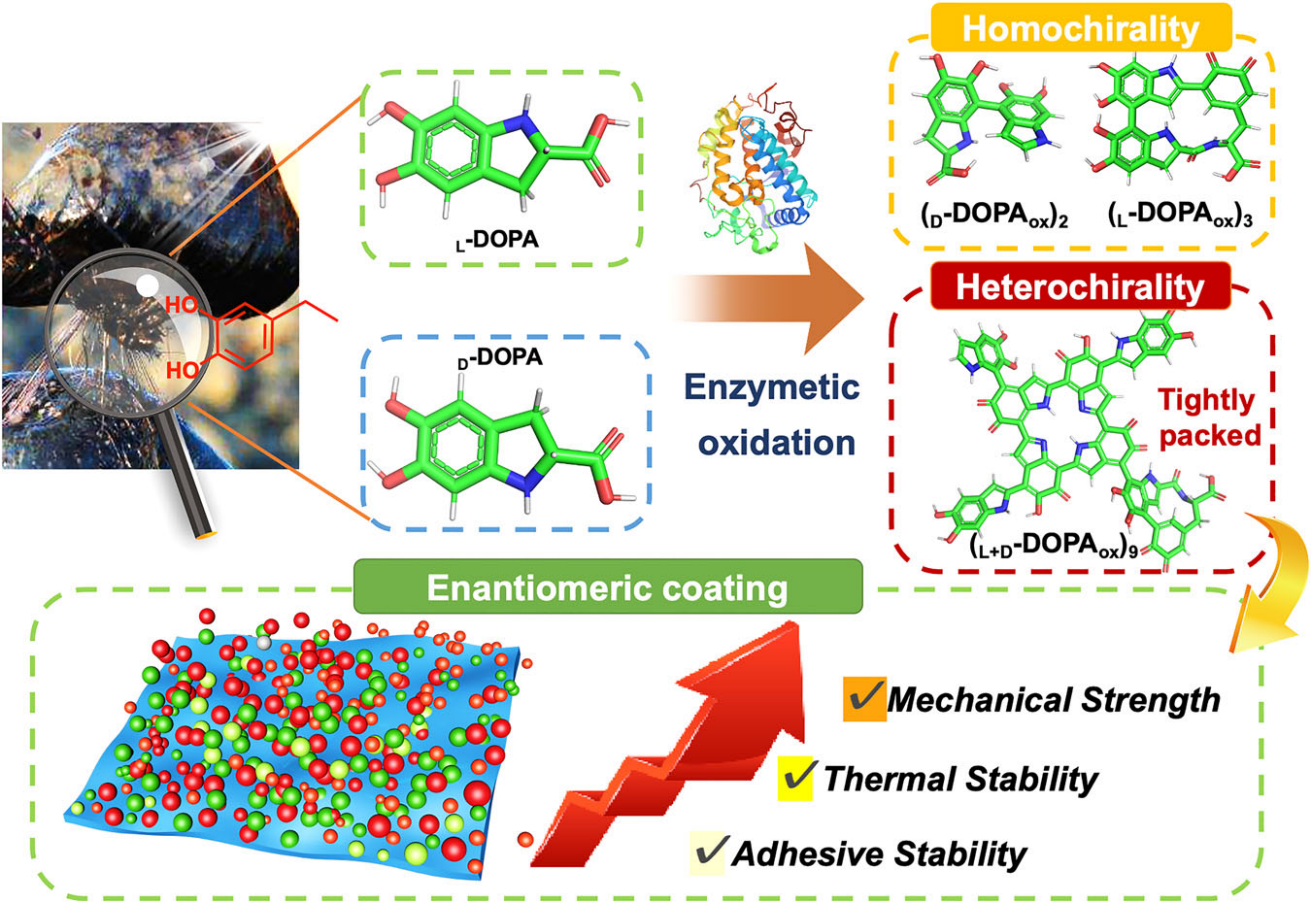
Schematic illustration of the enzymatic oxidation of chiral DOPA molecules inspired by natural mussels to generate different polymerization states and enantiomeric coating with excellent properties. The structure of tyrosinase from Agaricus bisporus is derived from PDB files (PDB ID: 2Y9X).

Here, we investigate the enzymatic-induced polymerization of chiral DOPA and explored the effect of monomer chirality on the properties of assembled polymer materials. The chirality can significantly alter the polymerization and assembly kinetics of DOPA and the morphology of the resulting nanostructures. The _L_-DOPA and _D_ -DOPA copolymerizes into larger molecular weight oligomers which can assemble into highly ordered layered stacking structures, leading to the formation of biomimetic films with high crystallinity and superior performance (Fig. 1). Surface-interface interaction studies confirmed the increased adsorption capacity and higher stability of the racemic _L+D_-DOPA coating, along with higher molecular-substrate adhesion, which further demonstrates the difference in the self-assembly effect of homochirality and heterochirality systems. Furthermore, the resulting racemic polymer coating exhibits enhanced thermal properties, and the _L+D_-composite has a significantly increased surface Young’s modulus compared to chiral monomers. Therefore, by changing the chirality to control the enzymatic polymerization and adhesion process, better-performing materials can be fabricated from _L+D_-racemic mixtures, which are attractive candidates for future nanotechnology applications.

## Results

### Influence of chirality on the enzymatic polymerization of DOPA

**Fig. 2:**
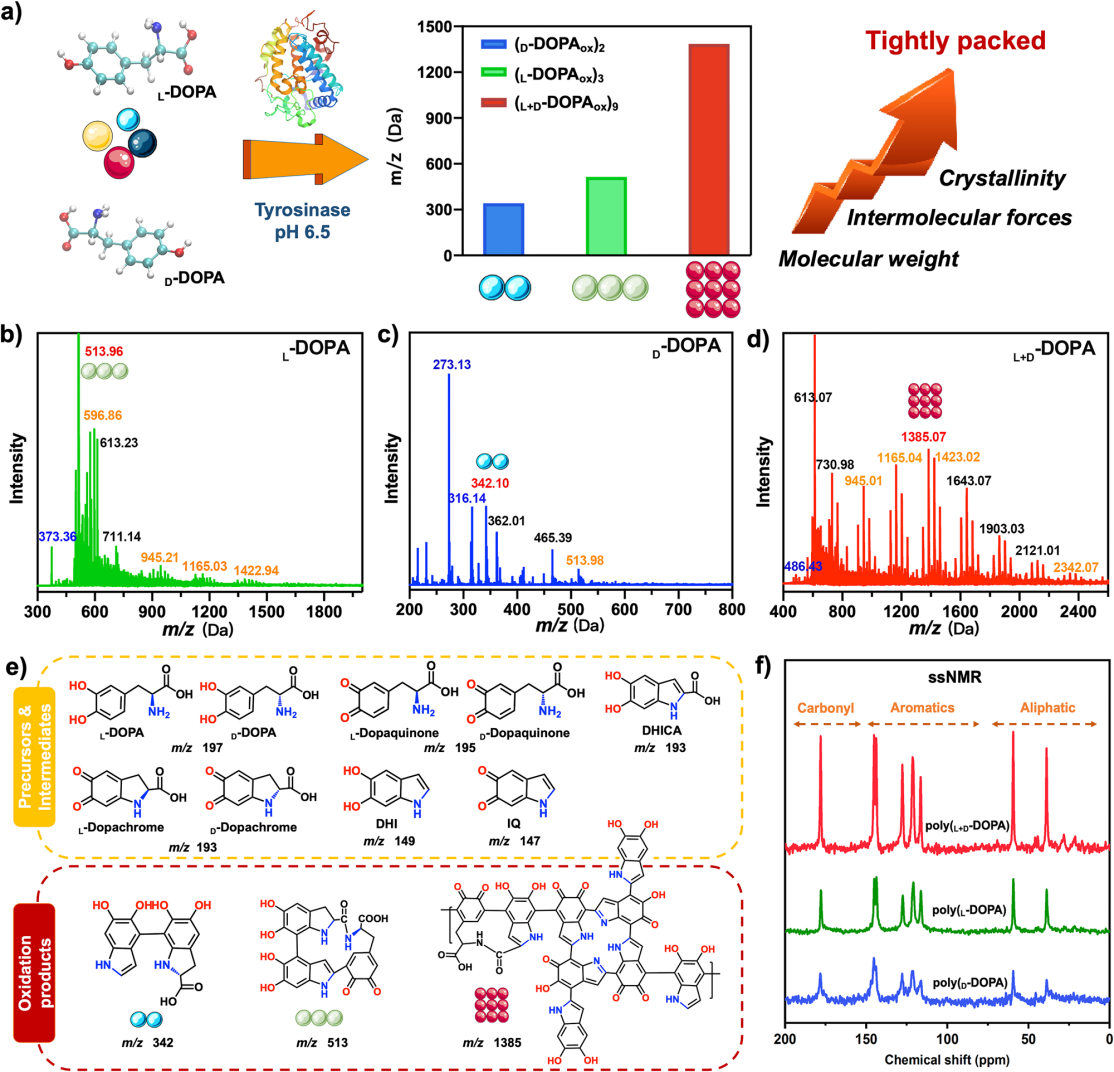
Chemical and molecular structural characterization of the enzymatic oxidation products of chiral DOPA substrates. **a** Schematic diagram of the enzymatic oxidation of chiral DOPA, in which the poly(_L+D_-DOPA) has a higher molecular weight, intermolecular interaction force and crystallinity, leading to the formation of tightly stacked supramolecular materials. We represent poly(_L/D/L+D_-DOPA) using green, blue, and red polymeric balls, respectively. **b**–**d** MALDI-TOF-MS analysis of the oxidative products of _L_-DOPA, _D_-DOPA, and _L+D_-DOPA, respectively. The peaks marked in red are the presumed main oxidation products (**e**) and the orange ones are other possible products (Supplementary Fig. 4), and the blue-labeled peaks can be attributed to the matrix 2,5-dihydroxybenzoic acid (DHB). **e** The proposed basic building blocks for the formation of eumelanin and the possible structures assigned to the main peak detected by MALDI-TOF-MS, which correspond to the enzymatic oxidation products of _L_-DOPA, _D_-DOPA, and _L+D_-DOPA, respectively. **f** CPMAS ^13^C NMR spectrum (75 MHz) of poly(_L_-DOPA), poly(_D_-DOPA), and poly(_L+D_-DOPA), respectively.

To evaluate the effect of chiral configuration on the oxidative process, we selected _L_-DOPA, _D_-DOPA, and their 1:1 mixed racemic _L+D_-DOPA solution as substrates and use tyrosinase to induce the oxidation (see “Methods”, Fig. 2a). The change of solution color confirms the oxidative polymerization to obtain melanin-like materials, and both photographs and UV light confirm the faster oxidation rate and better oxidation effect of _L+D_-DOPA (Supplementary Fig. 1). Regarding the final product by oxidation of DOPA, most theories currently believe that its oxidation yields eumelanin, which is the most common type of naturally occurring melanin. There has been little knowledge of melanin structure and oxidation pathway, but the molecular blocks that comprise the polymerized structure, such as dihydroxyindole (DHI) and dihydroxyindole−carboxylic acid (DHICA), have been partially understood (Supplementary Fig. 2)^31–33^. In the Raman spectrum of the above DOPA oxidation products, structural peaks such as in-plane stretching of aromatic rings and C-H stretching vibrations appear, which are defined as the melanin signals (Supplementary Fig. 3a)^34,35^. Fourier Transform Infrared (FTIR) spectroscopy (Supplementary Fig. 3b) revealed peaks at 3200, 1610, 1515 and 1360 cm^−1^, consistent with natural melanin. In addition, the ionic doublet peaks appear at 1515 and 1610 cm^−1^, which are consistent with the proposed indole or indoline structures^36,37^.

To investigate the influence of chirality on the enzymatic oxidation of DOPA, we studied the enzymatic reaction products of _L_-DOPA, _D_-DOPA, and _L+D_-DOPA at the molecular scale. Mass spectrometry analysis by matrix-assisted laser desorption time-of-flight mass spectrometry (MALDI-TOF-MS) shows that the oxidation products of DOPA are oligomers^38–41^. Interestingly, the mass spectrum peaks show that _L+D_-DOPA molecules are oxidized and polymerized mainly into nonamers ((_L+D_-DOPA_ox_)_9_, *Mw* 1386), while polymers with molecular weights up to 2000 are also produced. However, the oxidation of _D_-DOPA and _L_-DOPA yielded more dimers ((_D_-DOPA_ox_)_2_, *Mw* 342) and trimers ((_L_-DOPA_ox_)_3_, *Mw* 513), respectively, and the molecular weights of the polymer products were lower (Fig. 2b–e and Supplementary Fig. 4). To further explore their structures, solid-state NMR (ssNMR) was performed^42,43^. A comparison of CPMAS ^13^C NMR spectra for poly(_L-_DOPA), poly(_D-_DOPA) and poly(_L+D-_DOPA) is shown in Fig. 2f, whose chemical shifts are consistent with those reported previously for eumelanin. Moreover, resonances corresponding to the two enriched protonated aromatic carbons of synthetic melanoids for poly(_L+D_-DOPA) are dramatically enhanced compared with the homochiral poly(DOPA).

We speculate that there is a difference in the binding of the chiral substrate to the active site of tyrosinase, resulting in a differential reaction polymerization process. To investigate the affinity and interactions of the binding process, we selected _L_-DOPA and _D_-DOPA as ligands and investigated the two pair docking in three systems, _L_-DOPA + _L_-DOPA (LL), _D_-DOPA + _D_-DOPA (DD) and _L_-DOPA + _D_-DOPA (DL), both ligands were docked simultaneously upon docking with the active site of tyrosinase and each of which underwent 10 independent repeated docking on AutoDock Vina^44^. Significant differences in the affinities of the different stereospecific conformations were observed, with DL being the easiest to bind to the enzyme and having smaller binding energy (Supplementary Fig. 5), i.e. _L+D_-DOPA docked more readily to the tyrosinase, which may be an important reason for its faster reaction rate. Representative docking results are further selected for visual analysis, and we found that the main intermolecular interactions that promote substrate binding to the active site in the three systems include hydrophobic interactions and hydrogen bonding interactions, as well as π–π stacking interactions and salt bridges. As shown in Supplementary Fig. 6a–c, the kinetic data and binding parameters show that _L+D_-DOPA binds tightly to the enzyme recognition site and that the monomer close to the copper ion in the DL system has more hydrogen bonding and hydrophobic interactions than in the LL and DD systems, making it easier to bind into the active pocket. Therefore, we infer that the complex interactions between residues in the enzyme active site and chiral monomers play an important role in enhancing the reaction rate and changing the polymerization form.

To further understand the structural basis for the high specific activity of _L+D_-DOPA at tyrosinase active site, the Michaelis constant (K_M_), maximum velocity of enzyme-catalyzed reaction (*V*_max_), and catalytic efficiency (*V*_max_/*K*_M_ ratio) parameters of _L_-DOPA, _D_-DOPA and _L+D_-DOPA were analyzed. The experimental results show that the catalytic efficiencies for the different substrates correlate with docking affinities and _D_-DOPA being the poorest substrate (K_M_ = 2.081 mM, *V*_max_ = 50.30 μM min^−1^). _L_-DOPA is more structurally adapted to the natural tyrosinase and exhibits smaller K_M_ values (0.575 mM) and faster reaction rates (*V*_max_ = 95.97 μM min^−1^). In the case of the racemic system, the substrate showed the highest catalytic efficiency (*V*_max_/K_M_ = 220.26) with tyrosinase, which is consistent with the results of molecular docking simulation with the highest affinity and catalytic efficiency (Supplementary Figs. 7 and 8 and Supplementary Table 1). It has been reported that _D_-DOPA can substitute _L_-DOPA as a cofactor of the hydroxylase reaction without competing significantly as an alternative substrate for DOPA oxidation^45^. Based on the above molecular docking results, we speculate that _D_-DOPA acts as a cofactor in the racemic system, facilitating the movement of the _L_-DOPA substrate into the enzyme binding site, while the closer interaction in the heterogeneous chiral system improves the binding efficiency of the substrate to the enzyme, ultimately resulting in different polymerization effects.

**Fig. 3:**
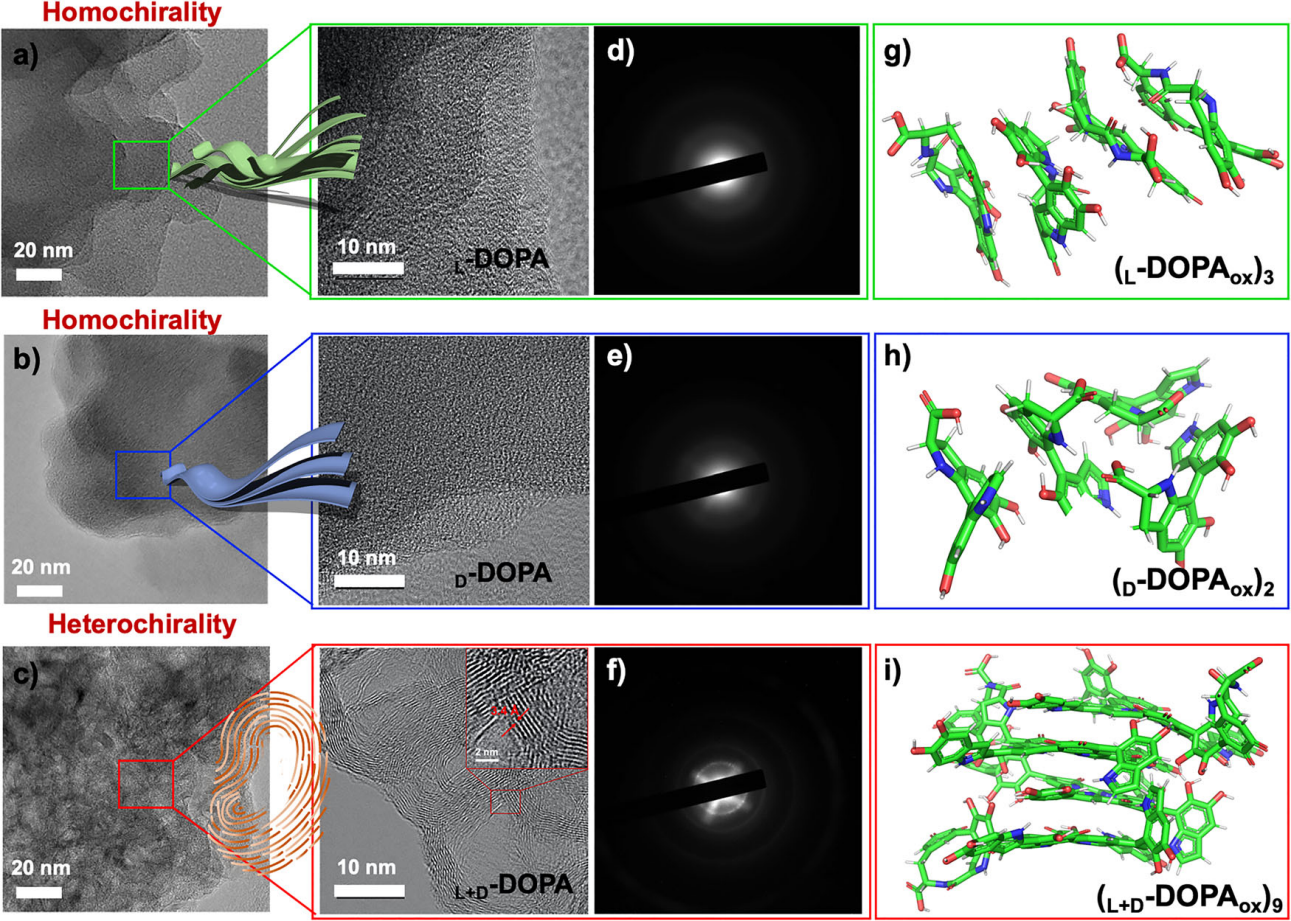
Micromorphological characterization and molecular dynamics simulation of three poly(DOPA) systems. **a**–**f** High-resolution transmission electron microscopy (TEM) (**a**–**c**) and selected-area electron diffraction (SAED) images (**d**–**f**) of the poly(_L_-DOPA) (**a**), poly(_D_-DOPA) (**b**), and poly(_L+D_-DOPA) (**c**) solutions. The scale bars are 20 and 10 nm for TEM. **g**–**i** Snapshot of the simulated aggregates constructed from DOPA oxidized polymers at the steady state of self-assembly.

The secondary structures of the three kinds of oxidized assemblies were characterized using circular dichroism (CD). Supplementary Fig. 9 shows that the mirror-image CD signals before the reaction of _L_-DOPA and _D_-DOPA gradually disappear with oxidation, while the _L+D_-DOPA solution always shows only very weak CD signals, indicating that the chiral secondary structure of monochiral molecules is destroyed with the reaction. The transmission electron microscopy (TEM) and selected-area electron diffraction (SAED) demonstrated that the poly(DOPA) using different chiral DOPA monomers forms stacked aggregates in solutions (Fig. 3a–c), which contain C, N, and O elements, as confirmed by the EDS elemental distribution analysis (Supplementary Fig. 10). The homochiral poly(_L_-DOPA) or poly(_D_-DOPA) tends to form disordered lamella structures without obvious crystal diffraction (Fig. 3a, b, d, e). Interestingly, the poly(_L+D_-DOPA) assembles into fingerprint-like nanostructures composed of stacked planes arranged in concentric rings (Fig. 3c and Supplementary Fig. 11), and the structures showed apparent diffraction spots in SAED analysis (Fig. 3f), indicating its higher crystallinity than that of the lamella structures assembled by homochiral poly(_L_-DOPA) or poly(_D_-DOPA). Synchrotron wide-angle X-ray scattering (WAXS) was performed to probe the molecular packing of poly(DOPA) within the self-assembled structures. All the poly(DOPA) solutions revealed three intense diffraction peaks at 2.27, 3.41 and 3.96 nm^−1^, indicating the formation of crystalline structures. The strong peak at 3.41 nm^−1^ is consistent with known π–π stacking distances for natural melanin^46^, and the higher peaks correspond to the layer-to-layer distance between poly (DOPA) laminar structures. The diffraction peak intensities of chiral molecules have obvious differences of poly(_L+D_-DOPA) >poly(_L_-DOPA) >poly(_D_-DOPA), which confirms that the racemic system has the highest crystal intensity and sheet stacking effect. (Supplementary Fig. 12).

Previous studies have provided a lot of evidence for the supramolecular organization in melanins, e.g. both synthetic and natural eumelanin are composed of planar sheets of varying dimensions, which are stacked to form nano-aggregates induced by noncovalent π–π stacking, electrostatic and solvophobic interactions^47,48^. Moreover, Kaxiras et al.^49^ predict that planar porphyrin-like oligomeric sheets of DHI and DHICA would be energetically stable and would stack in a staggered geometry. Based on the above characterization results and theoretical basis, we selected the most representative oxidation poly(DOPA) products, (_D_-DOPA_ox_)_2_, (_L_-DOPA_ox_)_3_ and (_L+D_-DOPA_ox_)_9_, as model blocks and carried out molecular dynamics simulations (MDSs) to calculate the self-assembling dynamics of poly(DOPA) molecules and their intermolecular interactions.

The square box systems with 150 poly(DOPA) molecules were established (Methods) and after energy minimization, all-atom molecular dynamics simulations were performed under the GAFF force field at constant temperature and pressure (Supplementary Fig. 13). The change of the root means square deviation (RMSD) of the molecule calculated by gromacs is shown in Supplementary Fig. 14, proving that the simulated systems have reached the equilibrium state. Among them, we selected representative structures for further discussion and analysis, and found that these poly(DOPA) molecules will quickly stack together to form a secondary structure through interactions such as π stacking and van der Waals forces to form larger structures (Supplementary Fig. 15). The MDSs results displayed in Fig. 3g–i show that the homochiral DOPA polymers, especially (_D_-DOPA_ox_)_2_, are more inclined to form isotropic materials, in agreement with the relatively random distribution of their aggregates, while the _L+D_-DOPA oxidation products are arranged parallel to each other, corresponding to their highly ordered layered structures observed in Fig. 3c. The experimental and simulation results jointly confirm that there are some layered stacking aggregates in the structure obtained by DOPA oxidation, in which the _L+D_-DOPA molecules are more ordered and packed tightly.

### Biomimetic coatings prepared by enzymatic oxidation and assembly of chiral DOPA molecules

**Fig. 4:**
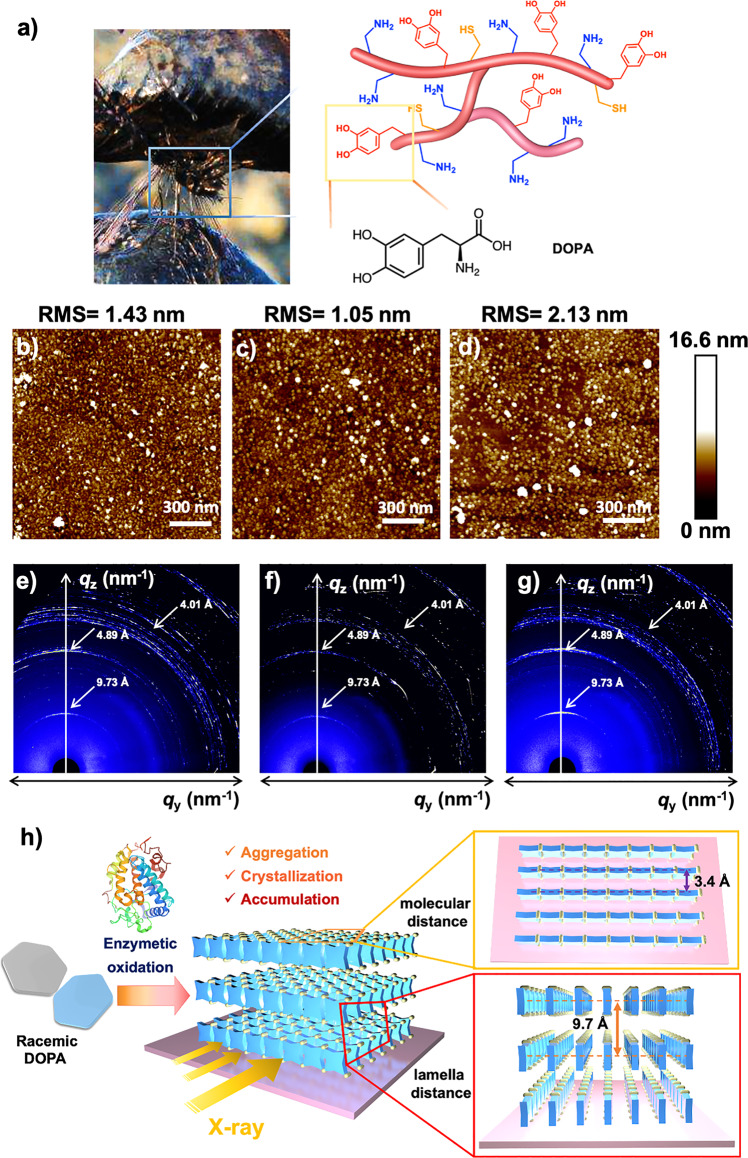
Preparation and characterization of poly(DOPA) films. **a** Photograph of the mussel byssus viewed from the base of the foot that distally attaches to the substratum and sequence of common mussel adhesion protein *Mefp-5*, among which DOPA, an important sequence that plays a role in adhesion, is enlarged by box selection. **b**–**d** AFM images of the surface modified by (**b**) poly(_L_-DOPA), (**c**) poly(_D_-DOPA), and (**d**) poly(_L+D_-DOPA). (Scanning area size = 1.6 × 1.6 µm, scale bar = 300 nm). **e**–**g** 2D-GIWAXS patterns of (**e**) _L_-DOPA, (**f**) _D_-DOPA, (**g**) _L+D_-DOPA polymers. **h** Schematic illustration of the preparation of poly(DOPA) film samples with inner layer-to-layer stacking, where the racemic system has the highest crystal strength and lamellar stacking effect.

Previous works have demonstrated that the _L-_DOPA is the vital motif for the underwater adhesion of mussels and other marine organisms, the strong affinity of DOPA molecules for substrates enables them to be immobilized on the surface through enzymatic polymerization to obtain uniform coatings (Fig. 4a, “Methods”)^50^. Atomic force microscopy (AFM) analysis found that uniformly distributed protrusions can be observed on the mica surface after coating, showing a granular surface morphology, demonstrating the successful loading of poly(DOPA) molecules on the substrate surface (Fig. 4b–d and Supplementary Fig. 16)^46^. The RMS roughness and average root mean square roughness (Rq) of the poly(_L+D_-DOPA) coating are both significantly higher than those formed by homochiral poly(_L_-DOPA) or poly(_D_-DOPA). It has been suggested that the coating formation is a sedimentation process of nano-aggregates synthesized in the reaction solution, and the deposition rate is closely related to the interaction between aggregates^51,52^. Dynamic light scattering (DLS) and scanning electron microscopy (SEM) were used to monitor the average size of aggregates in the reaction solution and the surface morphology of the resulting polymer coating, respectively. We found that the _L+D_-DOPA molecule has a smaller particle size, which is consistent with the SEM results and may indicate a denser intramolecular arrangement (Supplementary Fig. 17). The thickness and cross-sectional morphology of the poly(DOPA) film was observed using scanning electron microscopy (SEM) images (Supplementary Fig. 18a–c), showing the uniform dense areas adjacent to the substrates. SEM analysis revealed that the three self-assembled films have a regular cross-section, and their internal structures were all stacked and dense, with an average thickness of approximately 71.58 ± 1.2 μm, 68.38 ± 0.79 μm, and 72.65 ± 1.87 μm after multiple measurements of several parallel prepared films. In many groups of control samples, the thickness of the racemic poly (_L+D_-DOPA) system is slightly 2–5% higher than that of poly (_L_-DOPA) and poly (_D_-DOPA). At the same time, a denser internal structure can be observed in the magnified images, which confirms the higher adsorption capacity and tighter stacking of the _L+D_-system.

To know how assembled components are arranged in the solid state and structurally characterize the films self-assembled by homochiral and enantiomeric DOPA molecules, 2D grazing-incidence wide-angle X-ray scattering (GIWAXS) experiments were carried out on the poly(DOPA) dried on the silicon substrates. The films revealed similar diffraction peaks at 3.4–4 Å ascribed to strong benzene ring packing to those observed in solution polymerization. The anisotropic scattering image shows that the coating is oriented in the direction perpendicular to the substrate, and all of them have typical X-ray diffraction peaks, indicating the formation of crystalline structures, in which the film formed by _L+D_-DOPA has the highest degree of alignment and crystallinity (Fig. 4e–g and Supplementary Fig. 19). The peak at 9.73 nm^−1^ corresponds to a periodic reflection at 4.89 nm^−1^ perpendicular to the substrate surface, which probably results from the lamellar stacking of poly(DOPA) sheets one on top of another^53^. It’s consistent with the lamellar stacking morphologies obtained by TEM, and these data indicate that polymer molecules are orderly arranged in the plane of lamellar in both solution and film (Fig. 4h).

**Fig. 5:**
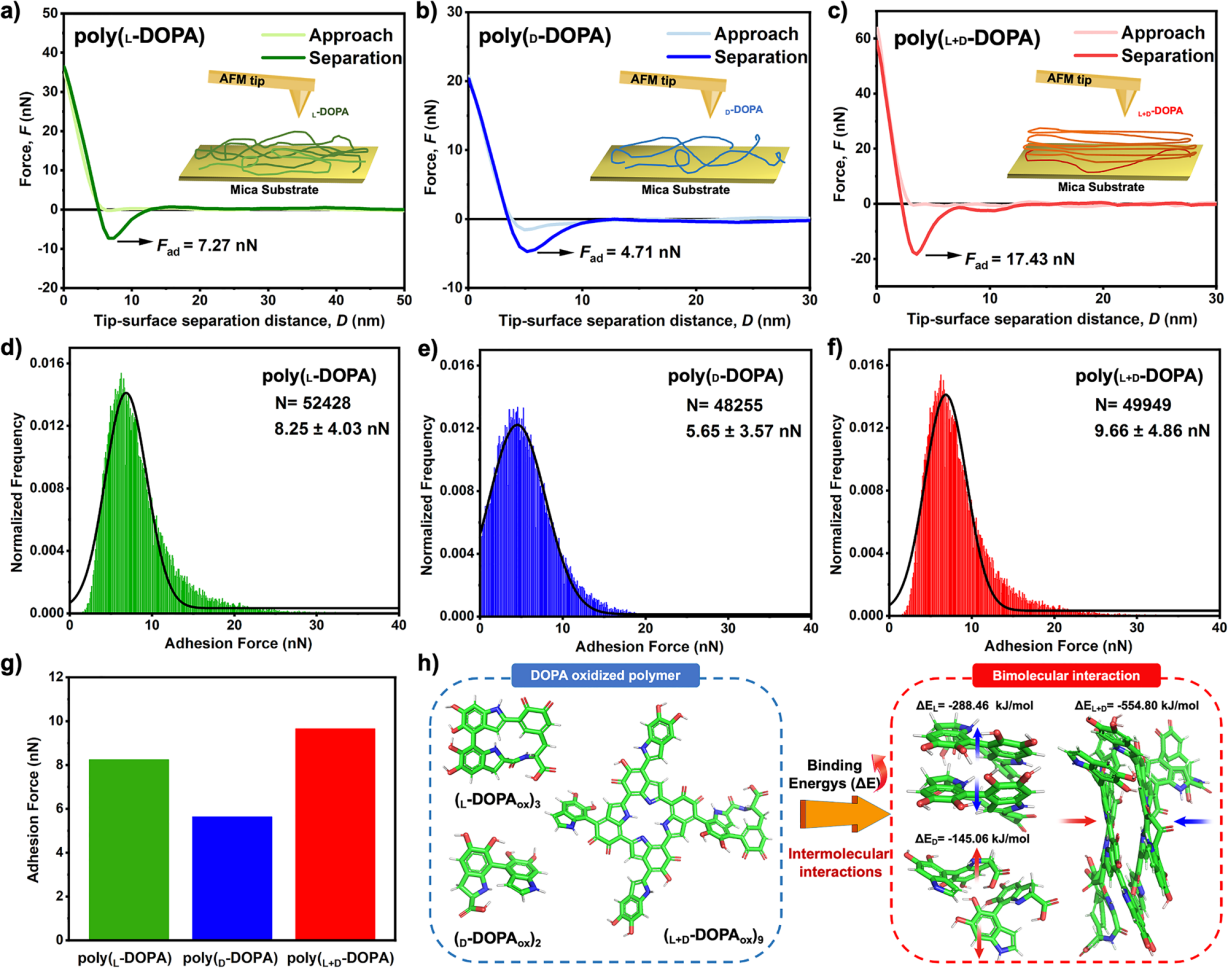
Intermolecular forces and binding energies analysis of chiral DOPA monomers. **a**–**c** Measured normal interaction forces of AFM tips with **a** poly(_L_-DOPA), **b** poly(_L_-DOPA), and **c** poly(_L+D_-DOPA) (the inset is the force curve measurement scheme.). **d**–**f** Adhesion distribution of the different surfaces coated with **d** poly(_L_-DOPA), **e** poly(_L_-DOPA), and **f** poly(_L+D_-DOPA). **g** Adhesive forces comparison of the poly(DOPA) films using different chiral DOPA monomers. **h** Schematic diagrams of the optimized geometric configurations of the three DOPA polymers obtained by DFT calculations and their binding energies during the two-molecule assembling process.

To further understand the effect of chirality on the adhesive properties of the poly(DOPA), the force curve measurements of poly(DOPA) were conducted using AFM^54^. Three chiral poly(DOPA) systems were deposited on mica substrates and then the AFM forces experiment was conducted as shown in Fig. 5a–c. In a typical force spectroscopy experiment, the silicon tip approaches the surface at a constant velocity, which retracts after reaching the constant peak force. The adhesive interactions between AFM tips and DOPA assemblies were shown in Fig. 5d–f, and the viscous force due to the tensile rate is also included. The adhesive force of poly(_L_-DOPA) on AFM tip (8.25 ± 4.03 nN) is higher than that between poly(_D_-DOPA) and AFM tip (5.65 ± 3.57 nN). However, the poly(_L+D_-DOPA) displayed the highest adhesion of 9.66 ± 4.86 nN compared to the monochiral systems. In addition, the representative F-X curves and rupture adhesive force distributions are shown in Fig. 5g.

Figure 5h shows the fully optimized geometries of the three DOPA polymers stabilized on the potential energy surface as determined by DFT calculations at the b3lyp/6-311G* level. According to the previously reported method^55,56^, the binding energy (E) of its two-molecule aggregation process was calculated, and it was found that the binding energy ΔE of (_D_-DOPA_ox_)_2_ (ΔE_D_) was −145.06 kJ/mol, and the ΔE of (_L_-DOPA_ox_)_3_ (ΔE_L_) was −288.46 kJ/mol, both far from lower than the binding energy of (_L+D_-DOPA_ox_)_9_ (ΔE_L+D_ = −554.80 kJ/mol), possibly due to the strongest benzene ring packing and intermolecular attractive forces in the heterochiral system.

### Chirality-dependent adsorption capacity and stability of poly(DOPA)

**Fig. 6:**
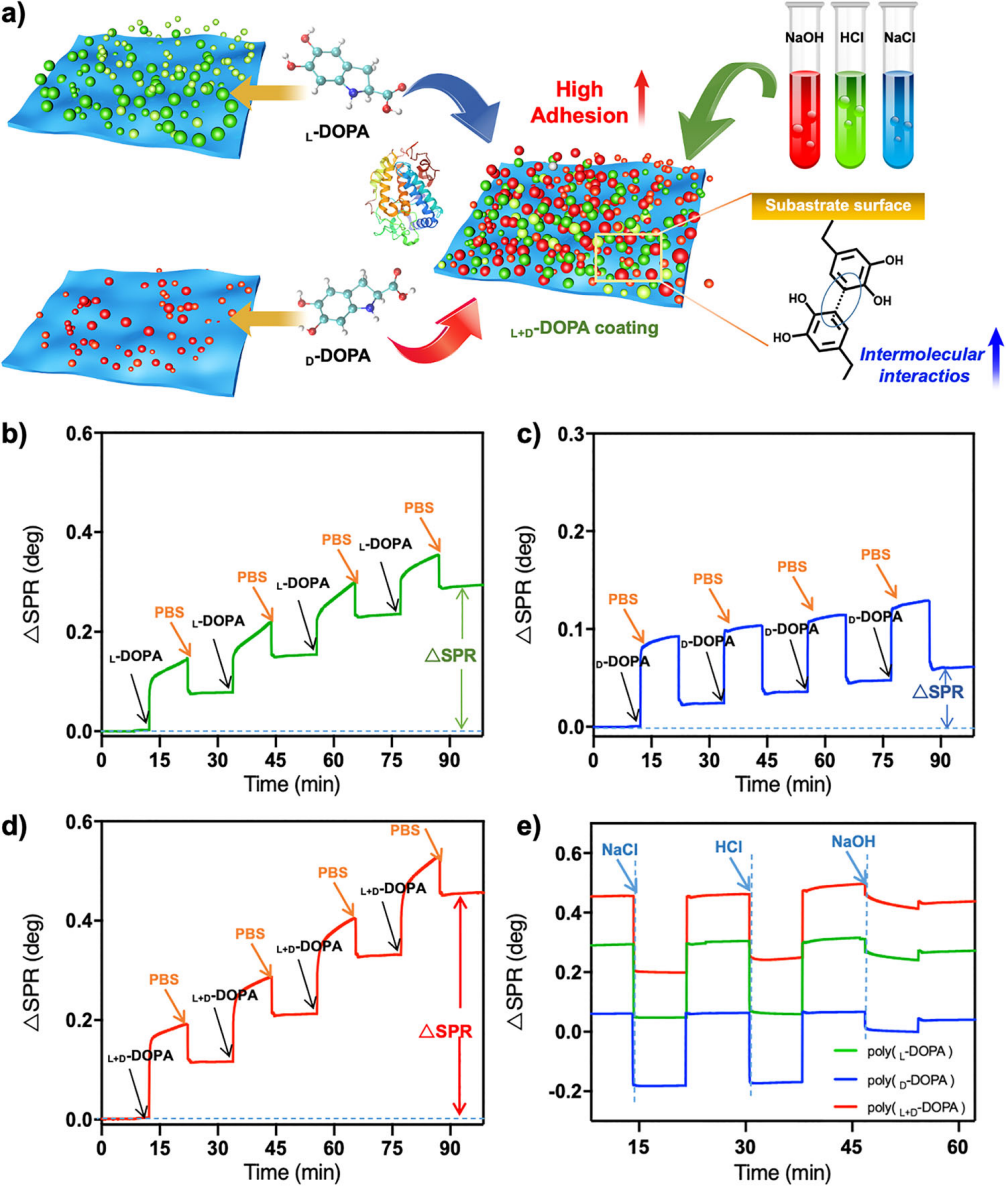
Characterization and adhesion stability of poly(DOPA) films. **a** Schematic diagram of the process of obtaining stable coating by DOPA chiral oxidation, in which the racemic system exhibits excellent stability. **b**–**d** The SPR sensorgrams of the oxidized **b**_L_-DOPA, **c**_D_-DOPA and **d**_L+D_-DOPA molecules adsorbed on the surface of bare gold chips. **e** Real-time monitoring of DOPA polymers-modified surfaces exposed to different solutions (0.01 M NaOH, 0.01 M HCl and 1 M NaCl).

The amount of poly(DOPA) absorbed on the gold surfaces was measured with surface plasmon resonance (SPR) by monitoring the change in the △SPR upon DOPA adsorption. As shown in Fig. 6b–d, with the injection of _L_-DOPA, _D_-DOPA or _L+D_-DOPA and tyrosinase, the SPR response value increased sharply to 0.119°, 0.086°, and 0.174°, respectively. The poly(_L+D_-DOPA) molecules adsorb on the surface of substrate at a high rate (about 103 ng cm^−2^ min^−1^), so its response speed of SPR is significantly faster than that of the homochiral poly(_L_-DOPA) or poly(_D_-DOPA) molecules (ca. 70 and 51 ng cm^−2^ min^−1^). Then, we injected DOPA molecules four times and found that the SPR response value of each modification of _L+D_-DOPA molecules was significantly higher than that of other homochiral DOPA, and finally the change of the SPR response is not obvious after injecting DOPA and washing with PBS again, indicating that the modified coating tends to be saturated and has excellent stability, and the adsorption amount of _L+D_-DOPA is nearly 2 times and 10 times higher than that of _L_-DOPA and _D_-DOPA, respectively. After assembly adsorption on the substrates, we found that the contact angle of the modified surface was significantly reduced (Supplementary Fig. 20), which was due to the hydrophilic properties of poly(DOPA), and the XPS detection confirmed that the poly(DOPA) films inherit the C, N, O elements from the monomers and the coating was uniformly loaded on the substrates (Supplementary Figs. 21 and 22). Compared with the homochiral DOPA system, after the adsorption reaches saturation, _L+D_-DOPA has a more pronounced decrease in water contact angle and higher C/N element loading, confirming its larger adsorption capacity, which is consistent with its larger thickness in the SEM analysis of the film section.

The stability of the coating films is one of the most important characteristics during their practical applications. We used SPR to test the stability of the oxidized DOPA films under strong acid, strong alkali, and high salt conditions. As shown in Fig. 6e, the strong adsorption of _L+D_-DOPA on the surface endows the polymer film with excellent acid and alkali resistance properties, showing better stability than the homochiral modified surface. We use the shedding rate (SR = (S_0_ – S_1_)/ S_0_, where S_0_ is the response value when the adsorption is stable, and S_1_ is the response value after the solution is eluted) to express the anti-elution stability of surface polymers. After washing with different solutions, the SR of _D_-DOPA modified surface was as high as 32.37%, and that of _L_-DOPA was 13.48%. The strong adsorption of _L+D_-DOPA_ox_ on the surface endows the polymer film with excellent stability, resulting in a shedding rate of only 10.25%.

### Chirality-dependent mechanical and thermal properties of poly(DOPA) films

**Fig. 7:**
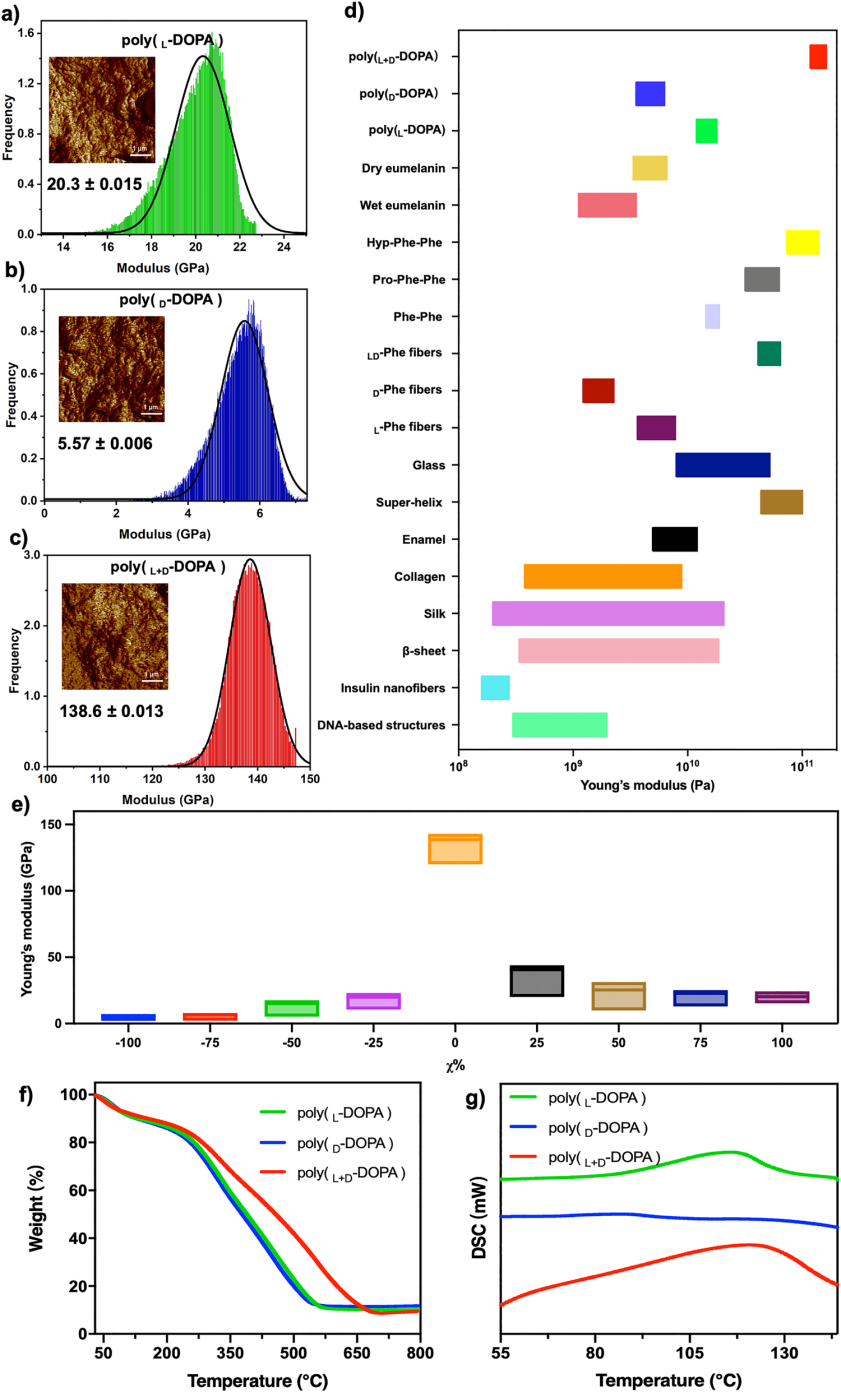
Mechanical properties and thermal stability of poly(DOPA) films. **a**–**c** AFM nanoindentation image and surface mechanical strength of poly(DOPA) films. **d** Comparison of surface Young’s moduli of different biological and non-biological materials. **e** AFM mechanical strength of poly(DOPA) films prepared by the enzymatic oxidation of DOPA with a different enantiomeric excess (χ%). **f**, **g** TGA spectra (**f**) and differential scanning calorimetry curves (**g**) of the poly(_L_-DOPA), poly(_D_-DOPA) and poly(_L+D_-DOPA), respectively.

We use an indentation-type AFM to measure the micromechanical properties of the homochiral and enantiomeric poly(DOPA) films. The elastic histogram and the value of surface Young’s modulus are shown in Fig. 7a–c^57^. Typically, the cantilever approached the surface of the films and retracted at a constant speed, and surface Young’s modulus was obtained by fitting the force-distance traces with the Hertz model. The measured surface elasticity of the poly(_L_-DOPA) film showed a surface Young’s modulus of 20.3 ± 0.015 GPa along the elongated direction (Fig. 7a), and the poly(_D_-DOPA) film showed a surface Young’s modulus of 5.57 ± 0.006 GPa (Fig. 7b). However, the poly(_L+D_-DOPA) film displayed a highly increased surface Young’s modulus of 138.6 ± 0.013 GPa (Fig. 7c), which was much higher than the 5.0 ± 1.7 GPa of natural eumelanin. In previous studies, the surface Young’s modulus of the Phe-Phe dipeptide^58^ and Hyp-Phe-Phe tripeptides was found to be about 19 to 102 GPa, which is derived from their unique super-helical organization and the enhanced inter-sheet interaction form hydrogen bonding^59^. As mechanical properties are directly correlated with higher-order packing of molecules, these data indicated a strong molecular packing within the poly(_L+D_-DOPA) film compared to the homochiral films, resulting in the formation of rigid materials displaying sufficiently high mechanostability (Fig. 7d). To better illustrate the effect of chirality on the coating properties, we further investigated the surface mechanical properties of poly(DOPA) coatings prepared by the enzymatic oxidation of DOPA enantiomers with different enantiomeric excess values (χ%) and the results are summarised in Supplementary Figs. 24–26 and Fig. 7e. The pure _D_-DOPA enantiomers (enantiomeric excess χ = −100%) with the lowest crystallinity and orderliness were measure with the lowest mechanical strength. With the increase of χ from −100 to 0, the mechanical properties of the coatings show an increasing trend and reach a maximum at the χ = 0, i.e. the racemic state. The surface mechanical strength of the system then falls back when the χ is further increased from 0 to 100. The results indicated that the chirality of the DOPA substrates has a great influence on the property of the coating films. As demonstrated in previous studies^60,61^, the multi-scale coverage from the atomic scale to the micron scale in the hierarchical architecture partly results in the toughening and strengthening mechanisms. We speculate that the highly ordered layer-by-layer structures at the nano- and molecular scales in poly(DOPA) lead to the combination of dense covalent and hydrogen bonds, which further yields multilayer composites with high mechanical strength. Furthermore, we performed a series of indentation tests on the poly(DOPA) film surfaces and the penetration depth was kept at a 10 mN load, and the characteristic load-displacement curves of the samples demonstrate the good compressive strength and elastic properties of the poly(DOPA) films (Supplementary Fig. 23a–c). The analysis revealed that the general value of poly(_L+D_-DOPA) samples were E = 38.8 ± 5 GPa and H = 1.3 ± 0.5 GPa, higher than E = 28.5 ± 6 GPa, H = 0.168 ± 0.05 GPa (poly(_L_-DOPA)) and E = 1.18 ± 0.4 GPa, H = 0.14 ± 0.03 GPa (poly(_D_-DOPA)) in the monochiral systems (Supplementary Fig. 23d). This implies considerable surface mechanical stability of the poly(DOPA) film, laying the foundation for its potential application in the coating field.

The thermal stability of the poly(DOPA) with different chirality was studied by thermogravimetric analysis (TGA). The results demonstrated that both homochiral and enantiomeric poly(DOPA) are stable up to 300 °C. However, with a further increase in heating temperature, the poly(_L+D_-DOPA) exhibited higher thermal stability compared to the homochiral poly(DOPA) (Fig. 7f). Differential scanning calorimetry (DSC) was used to determine the thermal properties of the poly(DOPA) assemblies. Lu et al.^62^ have previously studied the co-crystallization of stereopolycarbonates. Mixing equal amounts of amorphous enantiomer polymers can obtain crystalline stereocomplex polycarbonates with significant changes in thermal properties. Similarly, neither *T*_g_ nor *T*_m_ was observed in the DSC trace of the homochiral poly(_D_-DOPA), and the poly(_L_-DOPA) just showed a smaller endothermic peak with ΔH_m_ = 4.89 J g^−1^, while an endothermic peak with a wide range and large area is found at 117 °C with ΔH_m_ = 15.11 J g^−1^ for enantiomeric poly(_L+D_-DOPA), confirming its higher crystallinity compared to the monochiral system (Fig. 7g).

## Discussion

In addition to the surface elastic modulus, the influence of molecular chirality on the bulk elastic modulus of poly(_L/D/L+D_-DOPA) films has also been further investigated. As shown in Supplementary Fig. 27, although they also exhibit the best properties of racemic systems (0.762, 0.421 and 1.417 GPa, respectively), they are far from the excellent surface elastic modulus measured in Fig. 7. We speculate that this difference is due to the formation of the chiral poly(DOPA) films is a rapid oxidative cross-linking process under the action of tyrosinase. The differential interaction forces would be generated in the direction of the horizontal and vertical substrates and lead to different molecular arrangements, which will affect the physical properties of the materials. This process is not only a simple molecular deposition movement, but simultaneously involves a complex oxidative polymerization reaction and ordered molecular accumulation, which is consistent with the confirmation of its layer-by-layer stacking arrangement through experiments and simulations. At the same time, the polymer molecules finally deposited on the surface of the film should have the highest degree of cross-linking, which may be the reason for its excellent surface Young’s modulus along the elongated direction.

During polymerization, previous studies have confirmed that the chiralities of the monomer and the catalyst strongly influence the polymerization activities and the product regiochemistry^63^. Stereoselective polymerizations of chiral monomers can be achieved in two different ways: chain-end control (CEC) and enantiomeric site control mechanism (ESC). In the former case, the chirality of the growing polymer chain end has an important influence on enantioselection, which works in conjunction with enantiomorphic site control and affects the selectivity of the next monomer insertion. While in the second case, the chiral catalyst determines the incorporation of the next monomer. Meanwhile, stereoselective polymerization plays an important role in both conventional covalent polymerization and supramolecular polymerization, and up to now various methods have been reported to construct different types of substrate molecules and stereochemical sequences in polymerization^64,65^. In the competitive assembly process of enantiomers and racemates, many studies have shown that heterochirality aggregation occurs preferentially at lower scales to produce thermodynamic stable states^66^, while racemic stacks are more stable than those formed from single enantiomers at higher scales^67^.

Based on the results of this work, we propose an enzymatic polymerization mechanism to explain the observed phenomena of racemic systems. Under the catalytic action of tyrosinase, the _L_-DOPA and _D_-DOPA substrate in the racemic system acts as a reactant and cofactor, respectively, producing a faster reaction polymerization effect. The polymerization of chiral DOPA monomers is synergized by the combination of the two mechanisms of chain-end control (CEC) and enantiomeric site control (ESC). Due to the smaller stereohindrance between heterochiral molecules, the intermolecular interactions promote their aggregation and the chain transfer to a greater extent, resulting in a greater tendency to produce high molecular weight polymers and a thermodynamically stable state. After that, the aggregates are further stacked at a higher-order packing. Compared with the pure enantiomers, the molecular interactions in the _L+D_-mixed system produce stronger molecular stacking with an alternating arrangement of aromatic surfaces, which results in the layer-by-layer deposition of a directionally arranged crystal structure.

In conclusion, we show that the chirality of the DOPA monomer has an important effect on its enzymatic oxidation, polymeric assembly and the performance of the biomimetic films. The enantiomeric polymerization of _L_-DOPA and _D_-DOPA significantly alters the degree of polymerization and the assembly dynamics of the resulting nanostructures. Compared with the homochiral _L_-DOPA and _D_-DOPA, the crystallinity, thermal stability, and mechanical properties of the coating films after oxidation of the mixed racemates were significantly improved. The poly(_L+D_-DOPA) composite film inspired by natural melanin materials has a highly ordered lamella stacking arrangement inside and a surface Young’s modulus of up to 138.6 GPa. In the system with different enantiomeric excesses χ, the mechanical properties of poly(DOPA) coating prepared by enzymatic oxidation show regular changes, which is the lowest in the monochiral system and increases synchronously with the proportion of enantiomers, and reaches the highest value at χ = 0. These findings highlight the importance of chirality in covalent polymerization and self-assembly and provide further guidance for the deliberate design and synthesis of highly functional materials.

## Methods

### Chemicals and materials

The _L_- and _D_-DOPA (≥98%, CP) were purchased from Sigma-Aldrich (Beijing, China). Hydrochloric acid (HCl), sodium hydroxide (NaOH), sodium chloride (NaCl), phosphate, and potassium chloride (96%, AR) were obtained from Tianjin Kemiou Chemical Reagent Co., Ltd. (Tianjin, China). Phosphate buffered saline (PBS, pH 7.4; 10 mM phosphate, 138 mM sodium chloride, and 2.7 mM potassium chloride) was used as a flowing buffer. The water used in all experiments was prepared by a three-stage Millipore Milli-Q Plus 185 purification system (Millipore Corp., Bedford, MA) and had a resistivity of 18 MΩ cm. The pH values of all solutions were measured with an MP220 pH meter (Mettler Toledo, Switzerland). All solutions were filtered using a 0.22 µm syringe filter before use. Phosphate buffered saline (PBS, pH 7.4; 10 mM phosphate, 138 mM sodium chloride and 2.7 mM potassium chloride) was prepared in our laboratory. The buffers’ pH values were measured using an MP220 pH meter (Mettler Toledo, Switzerland). Ultrapure deionized water was produced by a Milli-Q water purification system (Millipore Corporation, Billerica, MA, USA).

### Synthesis of chiral DOPA polymers

In a typical experiment, chiral _L_-/_D_-DOPA (χ = ±100%) was dissolved in PBS buffer (50 mM, pH 7.4) to a final concentration of 2 mg/mL. By mixing two solutions in a ratio of 1:1, a racemic _L+D_-DOPA (χ = 0%) solution is obtained. DOPA aqueous solution with different χ values ((e.g., ±75%, ±50%, ±25%) were also prepared. The χ values were determined by the following equation:

1 $${{{{{\rm{\chi }}}}}}=\left[\left(\frac{L-D}{L+D}\right)\times 100\right]\%$$

where L and D are the moles of _L_-DOPA and _D_-DOPA, respectively. For example, 750 μL _L_-DOPA solution mixed with 250 μL _D_-DOPA solution resulted in a DOPA solution with χ = +50%.

In total, 5 mL of the above three solutions and 10 U mL^−1^ tyrosinase were mixed evenly, and the reaction was stirred at 37 °C for 6 h to obtain full oxidation. The final black solid products were obtained by centrifugation and washed three times with distilled water.

### Preparation of chiral DOPA polymeric films

_L_-DOPA and _D_-DOPA were dissolved in PBS to a final concentration of 2 mg/mL, and _L+D_-DOPA solutions were prepared with χ values of 0, ±75%, ±50%, ±25%, respectively, by mixing the _L_-DOPA and _D_-DOPA PBS solutions at different volume ratios. After ultrasonic cleaning, the mica substrates (*Ф* *=* 10 mm) were put into a container, followed by adding a mixed solution of DOPA and tyrosinase (enzyme concentration 10 U mL^−1^), and reacting at 37 °C for 6 h. After the reaction, the coating was washed and dried, leading to the formation of uniform coating loaded onto the substrate with a thickness of ~70 μm.

### Substrate specificity and determination of tyrosinase kinetic parameters

The absorbance curves against time were determined at room temperature by following the increase in absorbance at 475 nm (ε = 3700 M^−1^ cm^−1^) accompanying the oxidation of the _L_-DOPA^68^. Three different substrates (_L_-DOPA, _D_-DOPA, and _L+D_-DOPA) were used at room temperature to determine their affinity towards tyrosinase. The enzymatic reaction rate (V) was measured in PBS buffer (pH 6.80, 0.05 M) at different substrate concentrations ranging from 0.01 to 2 mg/ml. The Michaelis–Menten constant (*K*_*M*_) and maximum catalytic velocity (*V*_max_) were determined according to the method of Lineweaver and Burk^69^.

### Force curve measured by AFM

The force measurements were conducted using a commercial AFM (MultiMode 8, Bruker Corporation) in tapping mode with a silicon cantilever with a triangular pyramid tip (RTESPA-300-30, Bruker Corporation). In order to ensure the accuracy of the downforce effect, the normal spring constant of the cantilever was calibrated and two kinds of tips with different spring constants (k = 9 N m^−1^ and 40 N m^−1^) were used for the monochiral system with smaller Young’s modulus and the racemic system with larger Young’s modulus. All AFM experiments were carried out at room temperature. In a typical experiment, the AFM tip is moved normally toward and eventually penetrates into the prepared DOPA-coated mica substrate surface (coating thickness of ~60 μm) then the tip is retracted away from the surface. The adhesive interactions are manifested as negative force values. NanoScope Analysis software (1.90, Bruker Corporation) and AtomicJ software (version 1.7.3)^70^ were used to analyze the force curves and adhesive forces.

### Density functional theory (DFT) calculations

Discovery studio2019 was used to construct molecular models of three DOPA multimers (https://www.ncbi.nlm.nih.gov/sra/?term=DOPA). After forming the input files using GaussView6.0, we used Gaussian16 A.03 to perform configuration optimization and frequency calculations for the three molecules under the b3lyp/6-311G* method. The final molecular structure is generated after the frequency calculation results are free of imaginary frequencies. The distance between the two interacting molecules after optimization was measured using PyMol software.

### Molecular dynamics simulations (MDSs)

The classical MD simulation was performed using the Gromacs 2019.6 software package^71^. All constructed molecules and models are structurally optimized using Gaussian16 A.03, and usable structural topology files were generated using acpype. Use Gromacs tools and VMD to view and analyze MD simulation results. The Gromacs utils gmx rms, gmx sasa were used to analyze the root mean square deviation (RMSD) and solvent accessible surface area (SASA) of the simulated structures. The models of (_D_-DOPA_ox_)_2_, (_L_-DOPA_ox_)_3_ and (_L+D_-DOPA_ox_)_9_ were generated using Gromacs 2019.6 to generate square boxes with side lengths of 8 nm, into which 150 molecules and water solvent were added. This procedure uses the SPC water molecule model and uses the conjugate gradient (CG) algorithm for energy minimization^72^. The effect of the charge is taken into account in the simulation, and the total charge of the monomers is all zero (electrical neutrality). The 60ns all-atom molecular dynamics simulation was performed using Gromacs 2019.6 under the GAFF force field at a constant temperature of 298.15K and a pressure of 1.013 bar. After the end, the system was proved to be in equilibrium by calculating the RMSD.

## Supplementary information

Supplementary Information

## Data Availability

The authors declare that all the relevant data supporting the findings of this study are available within the article and its Supplementary Information files. Source data are provided with this paper and all data is available from the corresponding author upon request. Source data are provided with this paper.

## Source data

Source Data
