# Supplementary material for: Enzymatic polymerization of enantiomeric L−3,4-dihydroxyphenylalanine into films with enhanced rigidity and stability
Source: Nat Commun. 2023 May 26;14:3054. doi: 10.1038/s41467-023-38845-3 (PMC10219960; doi:10.1038/s41467-023-38845-3)
Supplement: Supplementary file 1 — Supplementary Information [file 41467_2023_38845_MOESM1_ESM.pdf]

Supplementary Information

Enzymatic polymerization of enantiomeric L-3,4-dihydroxyphenylalanine into films with enhanced rigidity and stability

Yuhe Shen,[1,#] Rongxin Su,[1,2,3,#] Dongzhao Hao,[1] Xiaojian Xu,[1] Meital Reches,[4] Jiwei Min,[1] Heng Chang,[1] Tao Yu,[1] Qing Li,[1] Xiaoyu Zhang,[5] Yuefei Wang,[5,6,] Yuefei Wang,[1,2,7]* Wei Qi,[1,2,3,]**

[1] State Key Laboratory of Chemical Engineering, School of Chemical Engineering and Technology, Tianjin University, Tianjin 300072, P. R. China

[2] Tianjin Key Laboratory of Membrane Science and Desalination Technology, Tianjin 300072, P. R. China

[3] Collaborative Innovation Center of Chemical Science and Engineering (Tianjin), Tianjin 300072, P. R. China

[4] Institute of Chemistry, the Hebrew University, Jerusalem 91904, Israel.

[5] State Key Laboratory of Component-based Chinese Medicine, Tianjin University of Traditional Chinese Medicine, Tianjin 301617, China.

[6] Haihe Laboratory of Modern Chinese Medicine, Tianjin 301617, China.

[7] Key Laboratory of Polymeric Materials Design and Synthesis for Biomedical Function, Soochow University, Suzhou, 215123, China

[#] These authors contributed equally.

* Corresponding authors: wangyuefei@tju.edu.cn (Yuefei Wang), wangyf0622@tjutcm.edu.cn (Yuefei Wang), and qiwei@tju.edu.cn (Wei Qi)

Table of contents

SUPPLEMENTARY METHODS **3**

ULTRAVIOLET-VISIBLE SPECTROSCOPY OF REACTION SOLUTION **3**

SCANNING ELECTRON MICROSCOPY (SEM) **3**

TRANSMISSION ELECTRON MICROSCOPY (TEM) **3**

GRAZING-INCIDENCE WIDE-ANGLE X-RAY SCATTERING (GI-WAXS) **3**

IN SITU SYNCHROTRON WIDE-ANGLE X-RAY DIFFRACTION (WAXD) **4**

CIRCULAR DICHROISM SPECTROSCOPY (CD) **4**

ATOMIC FORCE MICROSCOPY (AFM) CHARACTERIZATION **4**

POWDER X-RAY SCATTERING MEASUREMENTS **5**

MEASUREMENTS OF CHEMICAL MODIFICATION BY SPR **5**

MATRIX-ASSISTED LASER DESORPTION-TANDEM TIME-OF-FLIGHT MASS SPECTROMETRY .. **6**

SOLID-STATE NMR EXPERIMENTS **7**

CONTACT ANGLE (CA) MEASUREMENT **8**

XPS ANALYSIS **8**

RAMAN SPECTROSCOPY AND DATA ANALYSIS **8**

FOURIER TRANSFORM INFRARED SPECTROSCOPY (FTIR). **9**

NANOINDENTATION TESTS **9**

CALCULATION OF INTERMOLECULAR BINDING ENERGY (⊿E) **9**

MOLECULAR DOCKING STUDIES **10**

SUPPLEMENTARY FIGURES **11**

SUPPLEMENTARY TABLES **34**

SUPPLEMENTARY REFERENCES **35**

Supplementary Methods

Ultraviolet-visible Spectroscopy of Reaction Solution

2 mg mL^{-1} L/D/L+D-DOPA solutions were prepared, and 10 U mL^{-1} mushroom tyrosinase was added to make the reaction happen. Then the solution was stirred at 37°C for 6 hours. The UV-Vis time-correlated spectra were recorded and analyzed by drawing 1 mL solution from each sample at different time intervals. All UV–vis spectra were recorded on a UH5300 UV–vis spectrophotometer (Hitachi, Japan).

Scanning electron microscopy (SEM)

10 μL of prepared DOPA assemblies were deposited neatly on a microscope glass coverslip and dried in air. Samples of chiral DOPA polymeric films deposited on the substrate were taken directly to photograph cross sections. All samples were sputter-coated with platinum using an E1045 Pt-coater (Hitachi High-technologies CO., Japan), and then imaged with an S-8100 field emission scanning electron microscope (SEM, Hitachi High-technologies CO., Japan) at an acceleration voltage of 3 keV.

Transmission electron microscopy (TEM)

The morphology of the self-assemblies was further assessed using a JEOL JEM-F200 transmission electron microscope (TEM, JEOL Ltd., Japan) operated at 200 keV. To prepare samples, 10 μL aliquot of DOPA assemblies were placed onto a 200 meshes carbon-coated copper grid, and air dried.

Grazing-incidence wide-angle X-ray scattering (GI-WAXS)

The GI-WAXS measurements were performed at beamline 1W1A of the Beijing Synchrotron Radiation Facility (BSRF, Beijing, China), with a Xenocs-SAXS/WAXS system. The X-ray

wavelength was 1.5493 Å and the distance from the sample to the detector was set at 438 mm. The chiral DOPA films were obtained by dropping them on silicon substrates and drying at 37 °C. The samples were irradiated at a fixed angle of 0.2°.

In situ synchrotron wide-angle X-ray diffraction (WAXD)

In situ X-ray scattering measurements were carried out at beamline 1W2A of the Beijing Synchrotron Radiation Facility (Beijing, China). The wavelength of the radiation source was λ= 0.154 nm. Mar165-CCD was set at 160 mm sample-detector distance in the direction of the beam for WAXS data collections.

Circular Dichroism spectroscopy (CD)

The secondary structure of samples was characterized by circular dichroism (CD) with a Jasco-810 CD spectrophotometer (Japan). A freshly prepared sample (25 µL) was loaded carefully in a cell with a path length of 0.1 mm (Precision Cells, Inc., USA), which was scanned at room temperature by subtracting the buffer background. The presented spectra represent an average of three scans between 300 nm to 180 nm with a step size of 0.2 nm and a speed of 100 nm/ s.

Dynamic light scattering (DLS)

The particle size distribution of the chiral DOPA aggregates was measured using Zetasizer Nano-ZS (Malvern Instruments Ltd., UK). The samples were diluted 20-fold in Milli-Q water, transferred into a 1 cm path-length quartz cuvette, and measured at 25 °C. All replicates were performed three times.

Atomic force microscopy (AFM) characterization

The morphology of the chiral DOPA coating prepared by the above method was imaged by AFM. The AFM (MultiMode 8, Bruker Corporation) was operated in tapping mode with a silicon tip (ScanAsyst-Air, Bruker Corporation) and the scanning area was 1.6 × 1.6 μm^2. NanoScope Analysis software (version 1.90, Bruker Corporation) was used to compute an average of the average root mean square roughness (Rq). The AFM (MultiMode 8, Bruker Corporation) was operated in tapping mode with a silicon tip (ScanAsyst-Air, Bruker Corporation) and the scanning area was 1.6 × 1.6 μm^2. NanoScope Analysis software (version 1.90, Bruker Corporation) was used to compute an average Rq. The surface roughness values were an average result obtained from 3 samples.

Powder X-Ray scattering measurements

Freshly prepared samples were centrifuged and washed, followed by being frozen in liquid nitrogen and dried in a lyophilizer to yield a white powder for X-ray diffraction. The sample was placed in a fixed stage and the data was collected on Smartlab X-ray Diffractometer with a solid detector. The X-ray source is a Philips high intensity ceramic sealed tube (3 kW) and the X-ray of the wavelength of 1.5405 Å is generated by copper radiation and nickel filtration. The spectra were averaged with multiple scans.

Measurements of Chemical Modification by SPR

Gold-coated chips (BioNavis Ltd., Finland) were cleaned using an ultrasonic cleaner (KQ-250E, KunShan Ultrasonic Instruments Co., LTD, China). The chips were then immersed in alkali piranha solution ($H_2O/NH_3/H_2O_2$ = 5:1:1) at 75 °C for 10 min followed by extensive rinsing with ultrapure water and N2 blow dry. The gold chips were placed in an ultraviolet (UV)/ozone cleaning device (PSD-UV4-Novascan, USA) for 1 h. These chips were then rinsed with anhydrous ethanol

and Milli-Q water, and subsequently dried with high-purity N_2 before use. To evaluate the effect of chirality on the chemical modification of DOPA molecules, we dissolved L-DOPA and D-DOPA in PBS to a final concentration of 2 mg/ mL, and prepared a ratio of 1:1 L+D-DOPA solution. An SPR Navi 200A instrument (BioNavis Ltd., Finland) equipped with a 670 nm laser as the light source was used to measure the real-time modification of the chiral DOPA molecules on the gold chips. For each experiment, the baseline signal was established by flowing PBS buffer solution over the chip surface at a flow rate of 50 μL/min for approximately 10–20 min, and recording the initial SPR value and angle curve of the unmodified chip. Prepare 10 U/ ml tyrosinase solution, mix DOPA with enzyme solution and shake well, inject it into the flow cell at a flow rate of 10 μL/min for 10 minutes, and then rinse with PBS buffer solution at a flow rate of 50 μL/ min for 10 minutes minute. The injection of the above-mentioned DOPA-enzyme solution is repeated several times until the ΔSPR no longer increases to ensure that the gold chip reaches adsorption saturation. Record the saturated adsorption amount at the moment and the SPR angle of the modified chip. After the chemical modification is complete, we separately configure 1 M NaCl, 0.01 M HCl, and 0.01 M NaOH solutions, and use the SPR sensor to test the acid, alkali and salt resistance of the chip after stable adsorption. Use PBS buffer solution as the mobile phase, and in the order of salt→acid→alkali, inject the solution into the flow cell at a flow rate of 10 μL/ min for 10 minutes to test the change in adsorption capacity to evaluate the stability of chemically modified coatings. The values reported in the main text and SI are according to the means and standard deviations of the measurements.

Matrix-assisted Laser Desorption-tandem Time-of-flight Mass Spectrometry

500 μL of 1 mg·mL^{-1} sample aqueous solution and 500 μL of saturated 2,5-Dihydroxybenzoic acid (DHB) solution were mixed uniformly to analyze the molecular weight of the synthesized

products. Pipette 1 μl of the spot target and let it dry naturally at room temperature. Bruker MALDI-TOF MS technique (UltrafleXtreme) was used to determine molecular weight.

Solid-State NMR Experiments

^{13}C cross-polarization magic-angle spinning (CPMAS) NMR measurements were performed at a typical 15 kHz (±20 Hz) spinning speed and with a recycle delay of 10 s between successive acquisitions to observe resonances from the various carbon moieties. Ramped-amplitude cross-polarization, [1]in which the proton field strength was varied linearly by ∼20–50%, was implemented to compensate for inhomogeneous radiofrequency fields across the sample and improve spectral observation of DOPA polymers moieties with varying molecular mobilities. Typical 1–2 ms ^{1}H spin-lock times were used to transfer magnetization from ^{1}H to ^{13}C nuclear spin baths, and high-power heteronuclear proton decoupling (90–185 kHz) was achieved using the TPPM or SPINAL composite pulse sequences.[2, 3] High-fidelity ^{13}C direct polarization (DPMAS) experiments were conducted at typical 15 kHz spinning speeds, with 100 s delays between successive acquisitions. Typically, 24 000–36 000 and 1500–2000 transients were collected for CPMAS and DPMAS experiments, respectively, on the natural abundance DOPA oxidized polymers. [4, 5]In previous studies, several groups have tentatively identified functional groups in synthetic and natural eumelanin from different sources by using high-resolution ^{13}C and ^{15}N solid-state NMR[1] in conjunction with established chemical shift trends, such as those containing complex nnitrogen-containing aromatic ring structures. In all cases, the chemical shifts were consistent with molecular structures that include open-chain methylene groups (30 ppm), oxygenated aliphatic carbons (CHnO, 60–80 ppm), aromatic and/or olefinic carbons (110−160 ppm), and carboxyls (COO, 170–173 ppm). There were no prominent spectral features that could be attributed to proteinaceous materials (C_α, ∼50 ppm) or phenolic moieties (58 and 150 ppm).

Resonances corresponding to the two enriched protonated aromatic carbons in the ^{13}C NMR results of synthetic melanoids in the presence of L+D-DOPA substrates are dramatically enhanced compared with the chain methylenes, and prominent aromatic resonances could be observed.

Contact angle (CA) Measurement

An OCA15EC optical contact angle measurement (DataPhysics Instruments, Germany) was used to measure the contact angles and evaluate the surface wettability of samples at atmospheric pressure and 50 % relative humidity. The chips (L-modified, D-modified, L+D-modified and bare substrate) were mounted on the platform. Next, 1μL water droplet was injected on the. The contact angle values were measured using the instrument, which can capture and analyze the curve profile of water droplets in the three-phase interface. In order to obtain a reliable value for the contact angle, the measurements were performed by placing water droplets at five different locations on the sample surface and averaging the obtained values. The values reported in the main text and SI are according to the means and standard deviations of the measurements.

XPS analysis

The XPS spectra of poly(DOPA) modified surfaces were acquired using a PHI5000VersaProbe spectrometer (ULVAC-PHI, Japan). Survey spectra were recorded from 0 eV to 1350 eV at 1.0 eV steps and 150 ms dwell times, and the C 1s, N1s, O 1s and Si 2p spectra were collected.

Raman Spectroscopy and Data Analysis

Raman spectroscopy was performed by using DXR Smart Raman Spectrometer (Thermo Scientific, USA). A spectrum of buffer was subtracted from that of each time point to remove the broad contribution of water near 1650 cm^{-1}, followed by the removal of the spectrum of a blank quartz tube.

Fourier Transform Infrared Spectroscopy (FTIR).

The secondary structure of L+D-DOPA assembly was characterized by FTIR. The FTIR spectra were collected with an average of 16 scans on a Bruker Vertex-70 Spectrometer (USA) with 4 cm^{-1} resolution in the range of 4000-400 cm^{-1}. For sample preparation, the sample was frozen in liquid nitrogen and lyophilized to give a dry powder. The dried powder was mixed with KBr (at a ratio of 1:10 (w/w)) and grounded into a fine powder, followed by pressing into the transparent disk. The blank KBr disk was scanned as background, which was subtracted from the sample spectra.

Nanoindentation tests

Nanoindentation tests were performed by a NHT nanoindenter (Anton Paar, Switzerland) with a Berkovich diamond indentor at the maximum indentation load of 10 mN. The loading and unloading rates were 100 mN/ min. A hold time of 10 s was set between the loading and unloading cycles. The hardness and elastic modulus values were determined from load versus indentation depth curves using the Oliver & Pharr method. Three indentations were made for the poly(L-DOPA), poly(D-DOPA), and poly(L+D-DOPA) films, respectively, to obtain the mean hardness and elastic modulus values.

Calculation of Intermolecular Binding Energy (△E)

Molecular interaction models are constructed in GaussView 6.0. First, the pm6 method plus dispersion correction (D3) is used to optimize the structure of the models, and then the density functional theory is used to apply dispersion correction (DFT-D3). Under the b3lyp/6-311G* method, the single-point energy (E_A) of the optimized models is calculated; then the single-point energy (E_B, E_C) of the two single molecules were calculated under the same method respectively, and the binding energy was calculated according to the following formula:

$$\triangle E = E_A - E_B - E_C \quad (1)$$

Molecular docking studies

The crystal structure of tyrosinase from Agaricus bisporus (PDB ID:2Y9X, its structure has been elucidated utilizing X-ray crystallography with a resolution value of 2.78 Å) and was retrieved from the RCSB Protein Data Bank **(http://www.rcsb.org/pdb)**.[6] Water molecules of crystallization, Co-crystallized ligands (tropolone molecules) and holmium ions were removed from the crystal structure of enzyme using Discovery Studio Visualizer software. Save the final structure as a PDB file. The Kollman united atom charges were assigned after adding all polar and nonpolar missing hydrogens. Non-polar hydrogens were merged to their corresponding carbons using AutoDock Tools (enantiomeric molecules, and the structure of the enzyme was saved in pdbqt format, which could be read by the AutoDock Vina program).[7, 8]

The docking was performed for the prepared pdbqt files in the preceding steps with the help of AutoDock tools and AutoDock vina 1.2.3 programs, and calculations were carried out on ligands and MT model. During docking, the vina scoring function was used, and the search grid was extended over the copper ions of the target protein with a grid size of 20 Å× 20 Å× 20 Å and a grid spacing of 0.375 Å. The Center of the grid maps for energy scoring was set as (19.368 A°, 3.005 A°, -93.77 A°), and was generated using AutoGrid. During docking, two ligands were flexible and the protein was held rigid and kept the default settings for the rest. At the end of docking, structures are ranked according to binding energy, and the binding free energy for each run is provided in the docking log (txt) file. The best-scoring docking complex model was selected to represent the most favorable binding mode predicted by AutoDock vina. Structures were visualized using PyMOL and Protein-Ligand Interaction Profiler to obtain binding interactions.[9]

Supplementary Figures

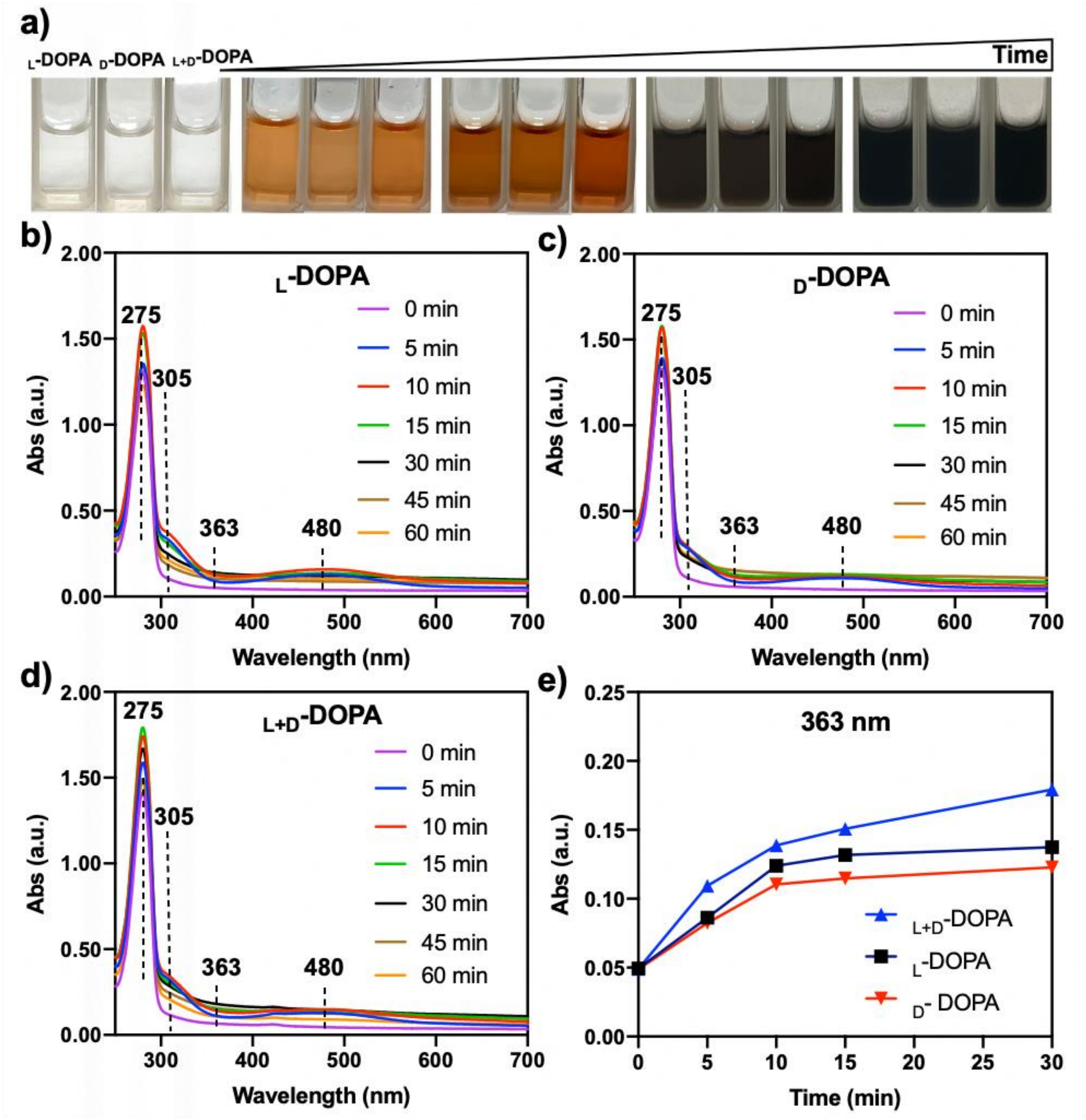

Supplementary Figure 1. Macrophotograph and UV–vis analysis of DOPA molecules. a) Macrophotograph and b-d) UV–vis spectra of the supernatant of DOPA molecules during

enzymatic oxidation at 37 °C. e) The kinetic curve of L-DOPA, D-DOPA and L+D-DOPA enzyme catalysis at 363 nm, which is reported as a characteristic peak of melanin intermediates.

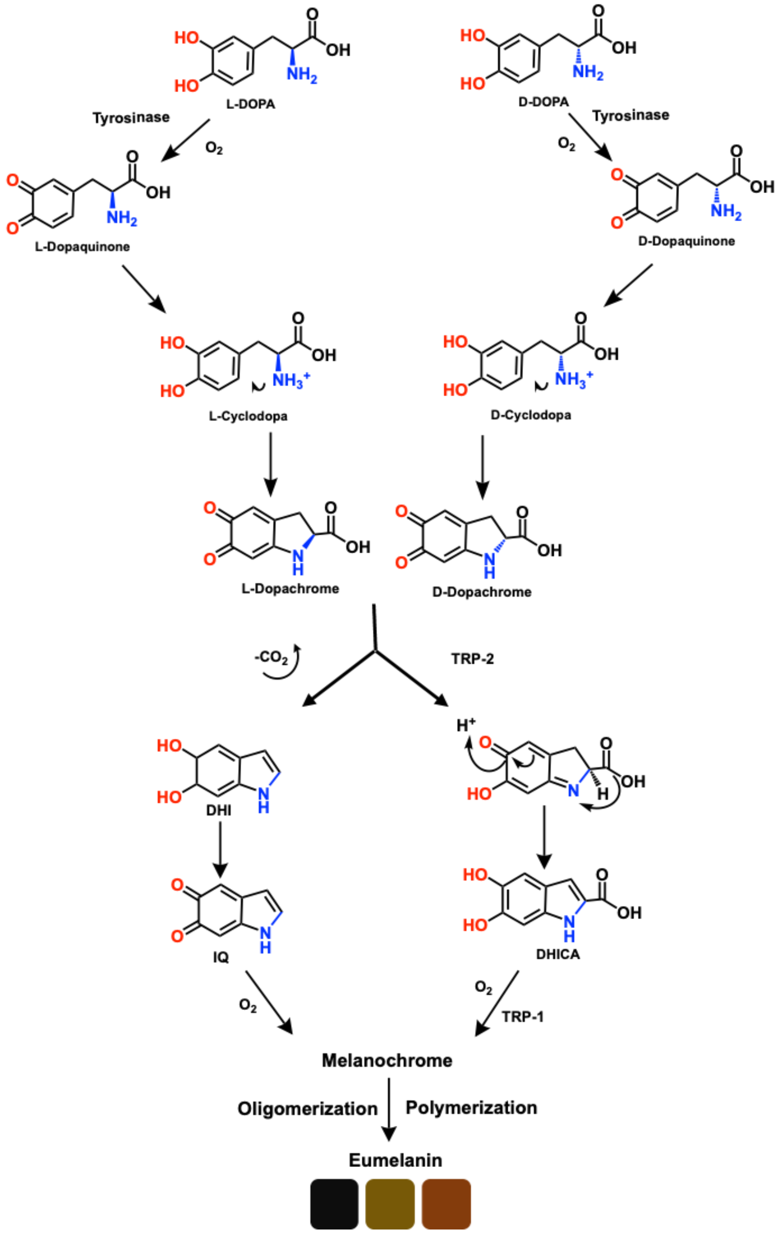

Supplementary Figure 2. Raper–Mason scheme of eumelanin production pathway using DOPA as a precursor. Both L-DOPA and D-DOPA get oxidized in the presence of the enzyme tyrosinase (TYR) to form dopaquinone (DQ), which readily undergoes cyclization and forms

dopachrome (DC) through the intermediate cyclodopa, which has a chiral configuration. Afterward, 5,6-Dihydroxyindole (DHI) or 5,6-dihrdoxyindole-2-carboxylic acid (DHICA) is produced from DC via decarboxylation or spontaneous rearrangement, respectively. The production of DHICA only happens in the presence of an enzyme called dopachrome tautomerase (DCT, also known as TYRP2). The thus-formed DHI and DHICA get oxidized to form the pigment precursor melanochrome (MC) and subsequently melanin.

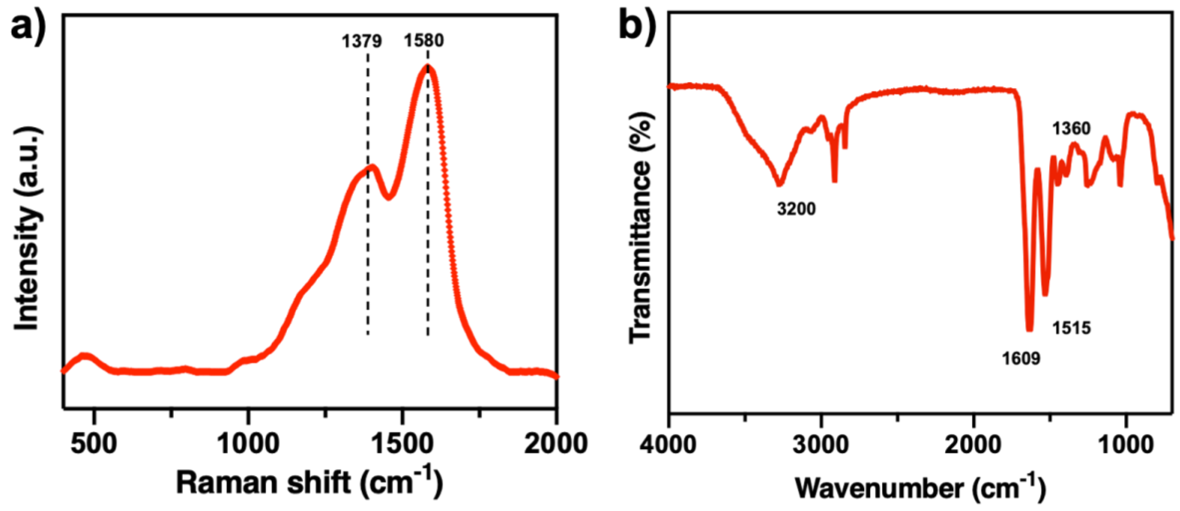

Supplementary Figure 3. Structural analysis of DOPA oxidation products. a) Resonance Raman spectra of the DOPA oxidation products, it shows that the peaks at 1379 and 1580 cm^{-1} were ascribed to the in-plane stretching of the aromatic ring (~1580 cm^{-1}), the linear stretching vibration of the C–C bond in the ring, and some C–H stretching vibrations from methyl and methylene groups (~1379 cm^{-1}), which are defined as the melanin signals. b) FT-IR spectroscopy of products formed after oxidative polymerization of DOPA. The large, broad peak spanning 3100–3300 cm^{-1} originates from the OH and NH stretching vibrations. The 1610 cm^{-1} may be caused by the combination of the C=O stretching of the carbonate group and/or the C=C aromatic ring vibration, and the 1360 cm^{-1} peak comes from indole ring vibration and/or CN stretching.

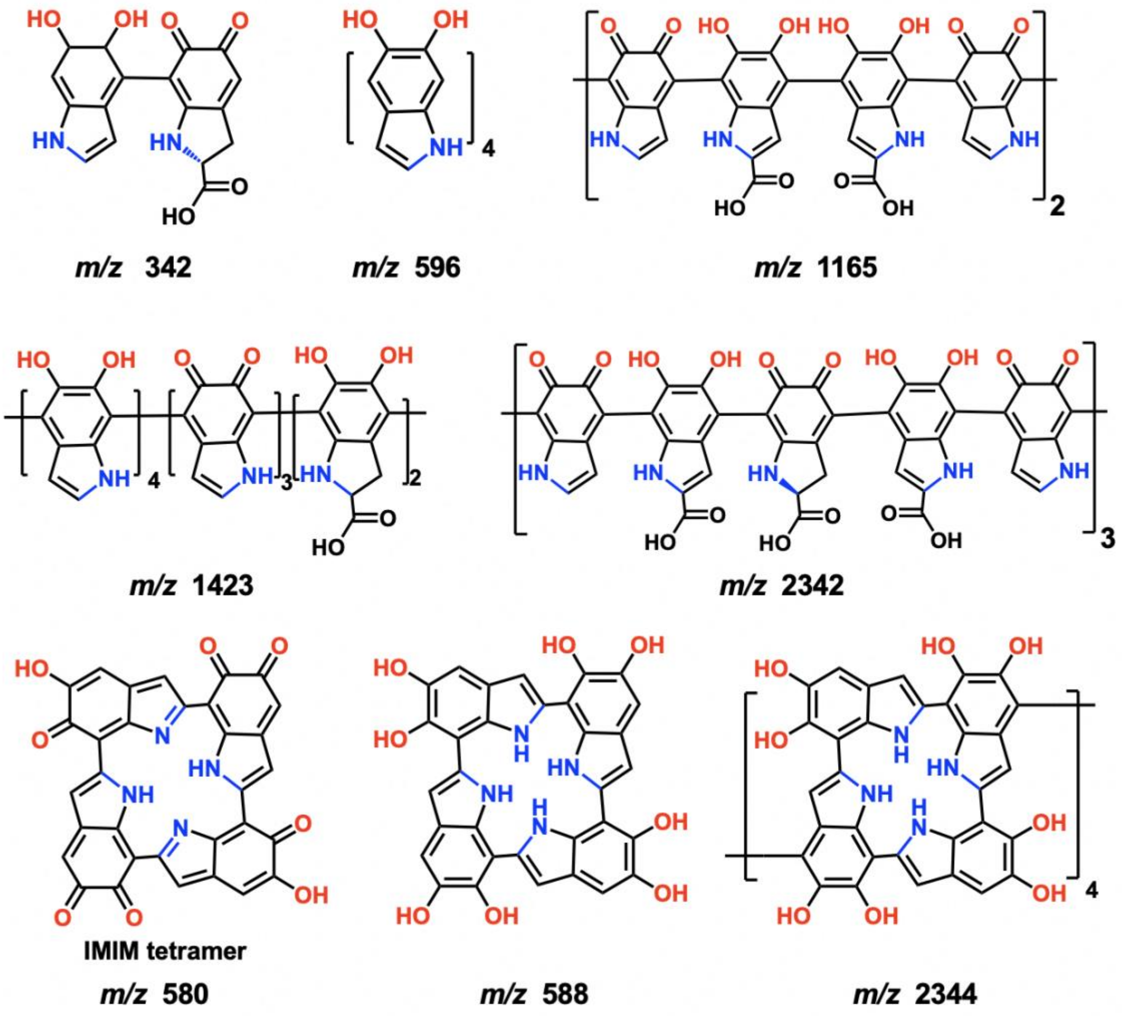

Supplementary Figure 4. Possible structures assigned to the other peaks marked in orange in Fig. 4e, which are detected by MALDI-TOF-MS. The derivation of each molecular formula is mainly based on the oxidation process and main building blocks of natural melanin reported in kinds of literature, among which the core motif of $(\text{L+D-DOPA}_{ox})_9$ is inspired by the IMIM tetramer, which is derived from the recently proposed structural model for eumelanin protomolecules.[10] It was confirmed by the combination of simulation and experimental results as an important reason for the lamellar structure in eumelanin.

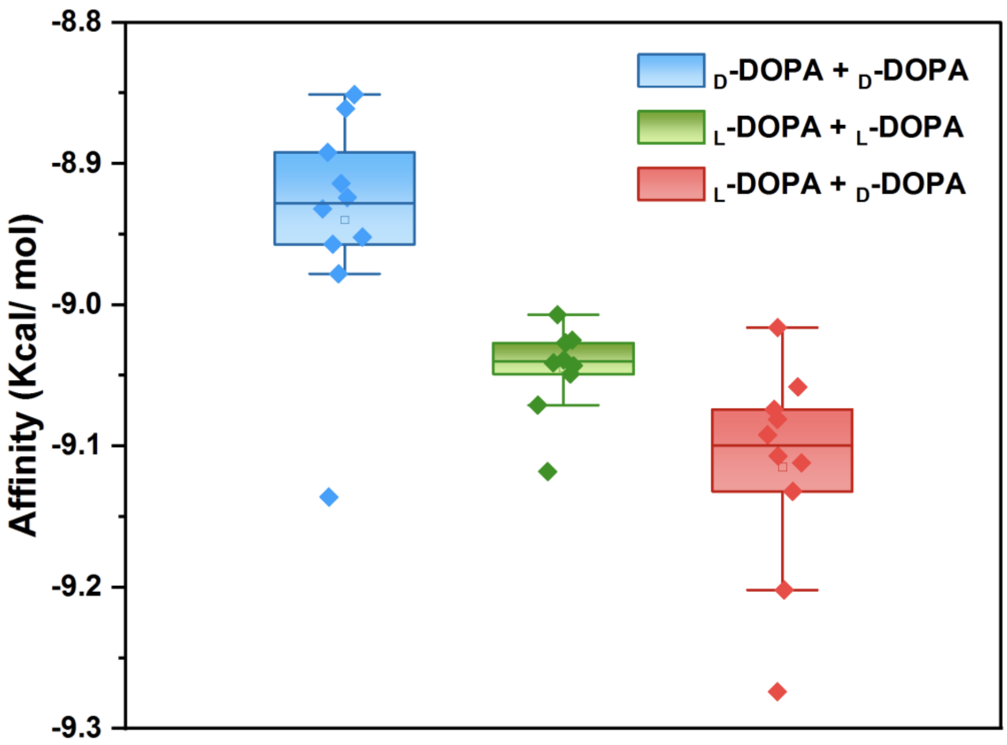

Supplementary Figure 5. Schematic representation of the results of ten independent docking of different chiral DOPA substrates with tyrosinase. Data are presented as mean ± SD (n = 10 independent test results). L-DOPA and D-DOPA were selected as ligands and docked simultaneously in three systems, L-DOPA + L-DOPA (LL), D-DOPA + D-DOPA (DD) and L-DOPA + D-DOPA (DL), both ligands underwent 10 independent repeated docking. Significant differences in affinity arise with the different stereo spatial conformations. We sorted out the docking energies obtained from ten docking operations under three conditions and plotted the box graphs. The statistical rule shows that DL has the lowest energy and binds most readily to the enzyme, and LL and DD have smaller binding energies, i.e. the two molecules with different chirality dock more readily to the tyrosinase, which may be an important reason for the faster reaction rate of L+D-DOPA.

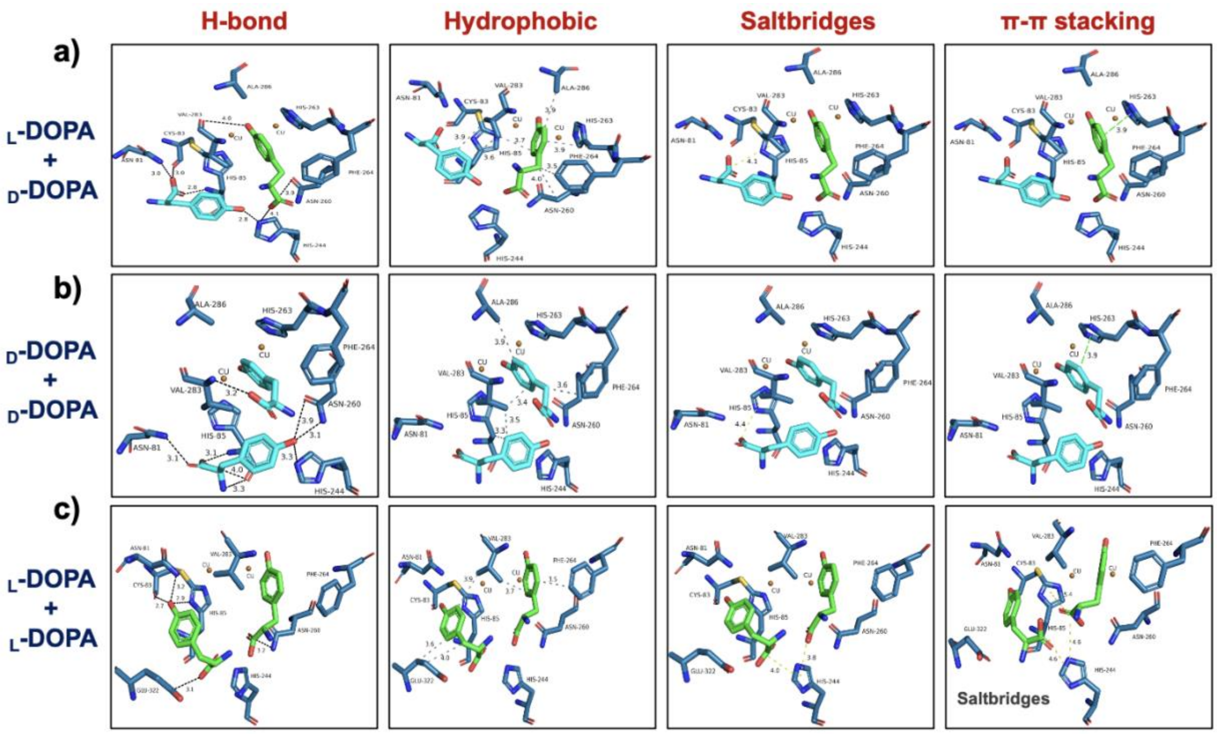

Supplementary Figure 6. Schematic representation of the interaction in the chiral DOPA substrate system with the active site of tyrosinase. In the diagram, the L-DOPA is labeled in green and D-DOPA is shown in Cyan, the interactions are marked by dotted lines of different colors (black: hydrogen bond, gray: hydrophobic interaction, green: π - π stacking, and yellow: salt bridge). The comparison of intermolecular forces of the three systems shows that the heterochiral molecules (a) have the strongest interactions, revealing the reason for the faster reaction of L+D-DOPA. Compared to the LL (c) and DD (b) systems, the DL system, in which L-monomer close to the copper ion, formed a large number of hydrophobic interactions with surrounding residues such as *ALA-286, HIS-263*, *PHE-264, ASN-260*, *VAL-283*, and *HIS-85*, while forming more hydrogen bonds with residues *VAL-283*, *ASN-260, and HIS-244*, which makes it easier to bind to the active pocket. D-monomer in the DL system does not directly participate in the reaction but interacts with surrounding residues outside the active pocket, which may affect the subsequent reaction process as a cofactor. It forms a total of four hydrogen bonds with residues *ASN-81*, *HIS-*

85, *ASN-260*, and *HIS-244*, which is lower than the seven hydrogen bonds in the DD system and is beneficial for the next monomer to enter the active site and react when the monomer close to the copper ion completes the reaction. For the DD and LL monochiral systems, we found that the salt-bridge interaction of the L-DOPA monomer with the *HIS-244* residue was generated when it closes in proximity to the copper ion in the LL system.

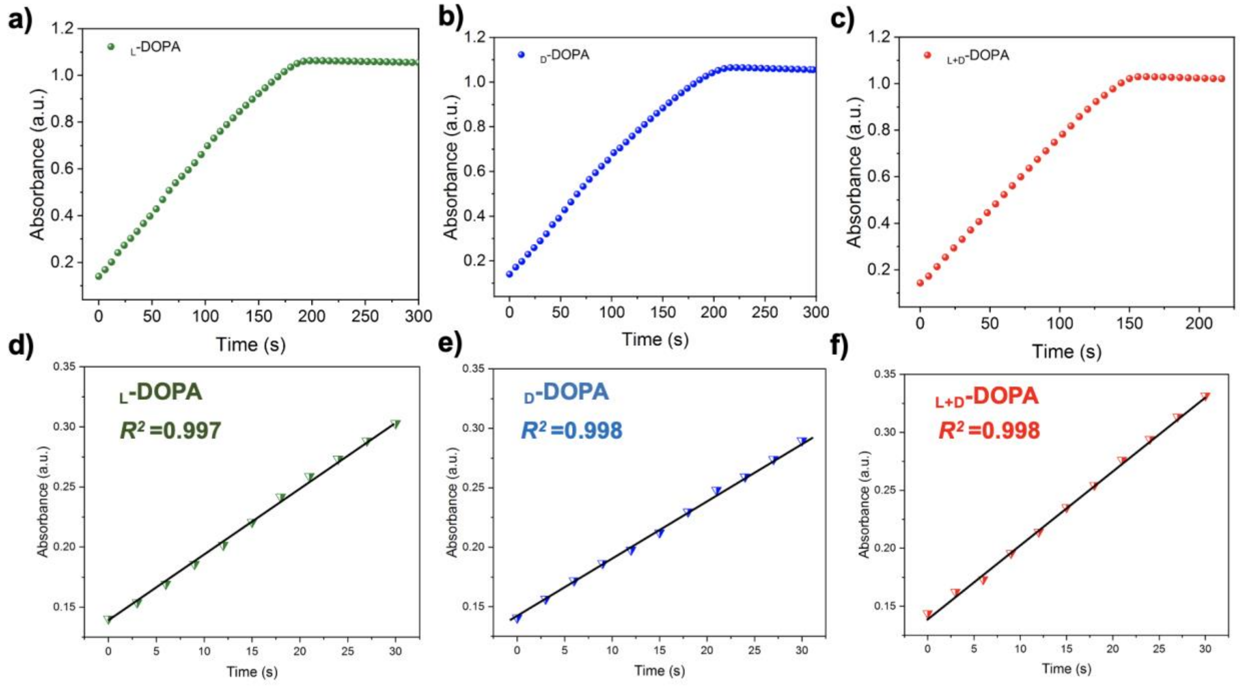

Supplementary Figure 7. Characterization of the catalytic kinetics of tyrosinase catalyzing different substrates. a-c) Reaction–time curves of poly(DOPA) colorimetric reaction catalyzed by natural tyrosinase. d-f) The magnified initial linear portion of the reaction time curve for tyrosinase-catalyzed DOPA oxidation.

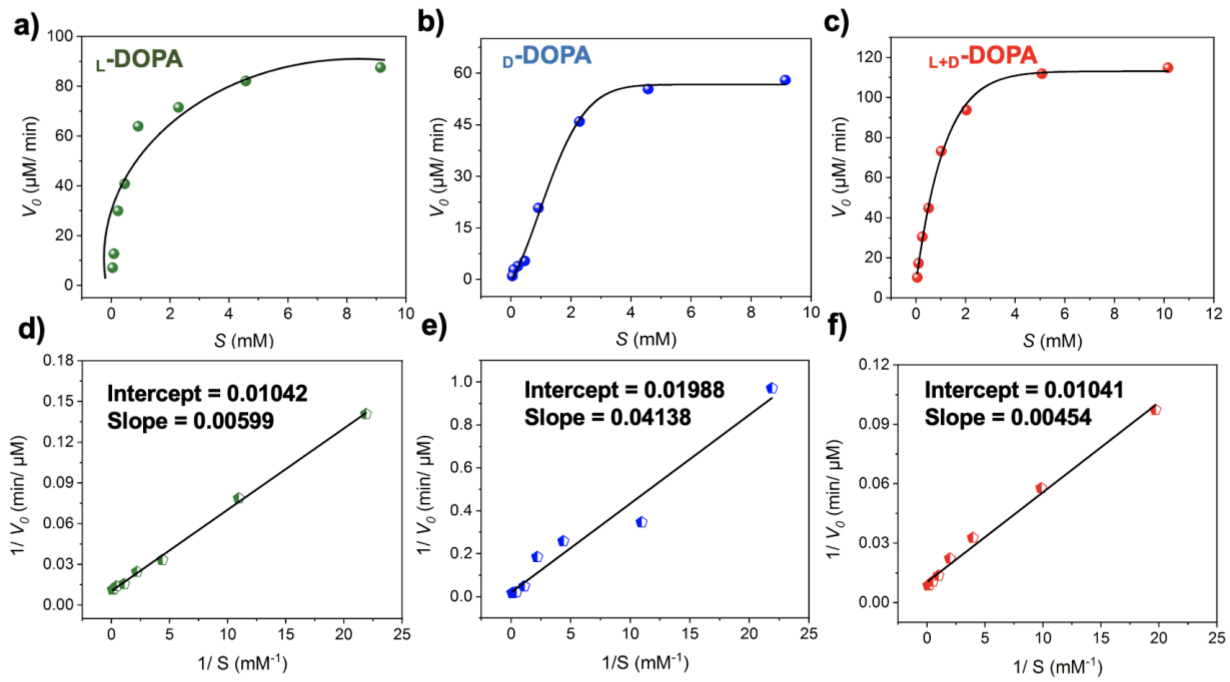

Supplementary Figure 8. Kinetics of oxidation at different concentrations of chiral DOPA substrates by tyrosinase. a-c) The plots of the initial reaction velocity versus DOPA concentration by the tyrosinase (S: substrate concentration, V_0： the apparent initial reaction velocity). d-f) Kinetic parameters of poly(DOPA) catalyzed by tyrosinase obtained using the Michaelis-Menten model by nonlinear regression method.

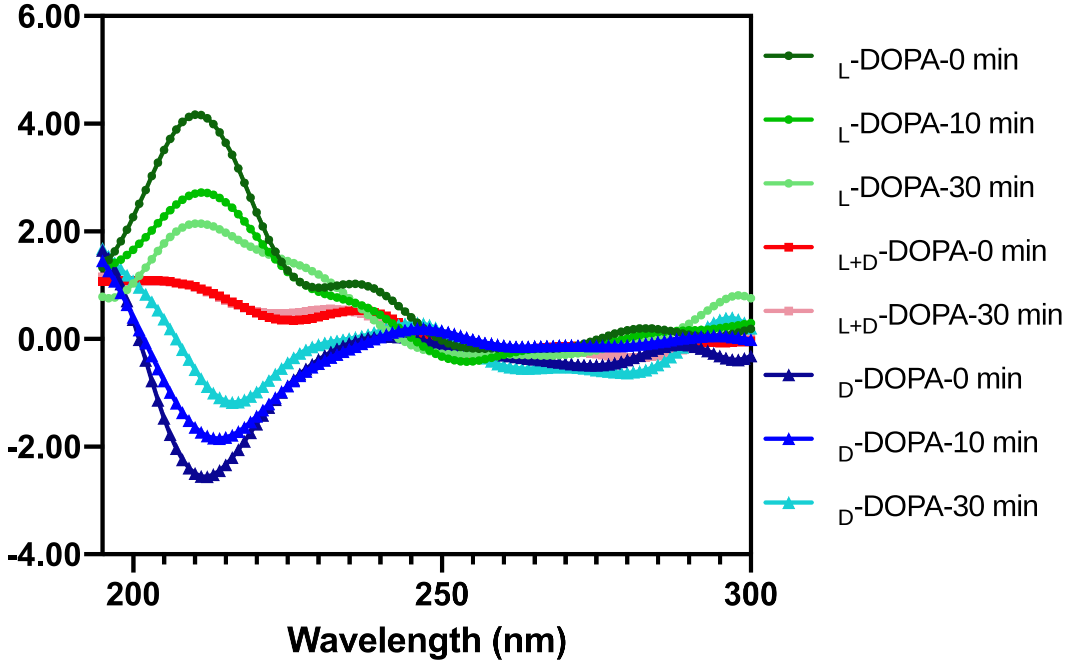

Supplementary Figure 9. CD spectra of self-assembled nanostructures of chiral DOPA molecules after different reaction times. Among them, the CD peak at 218 nm of L-DOPA (positive) and D-DOPA (negative) solutions may be attributed to the n-π* transition of amido bond. As the reaction progresses, the CD signals of the enantiomers are gradually weakened, but still appear as mirror images, and the racemic solution has no corresponding CD signal in this region.

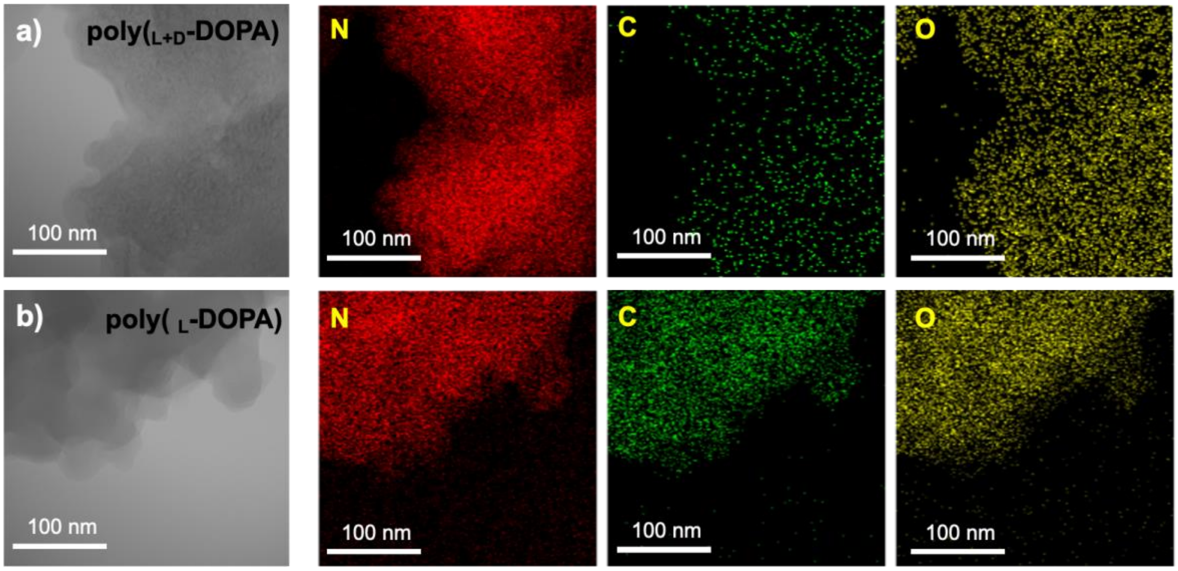

Supplementary Figure 10. Representative TEM images and EDS analysis of poly(L+D-DOPA) (a) and poly(L-DOPA) (b) aggregates in solution. The scale bars are 100 nm.

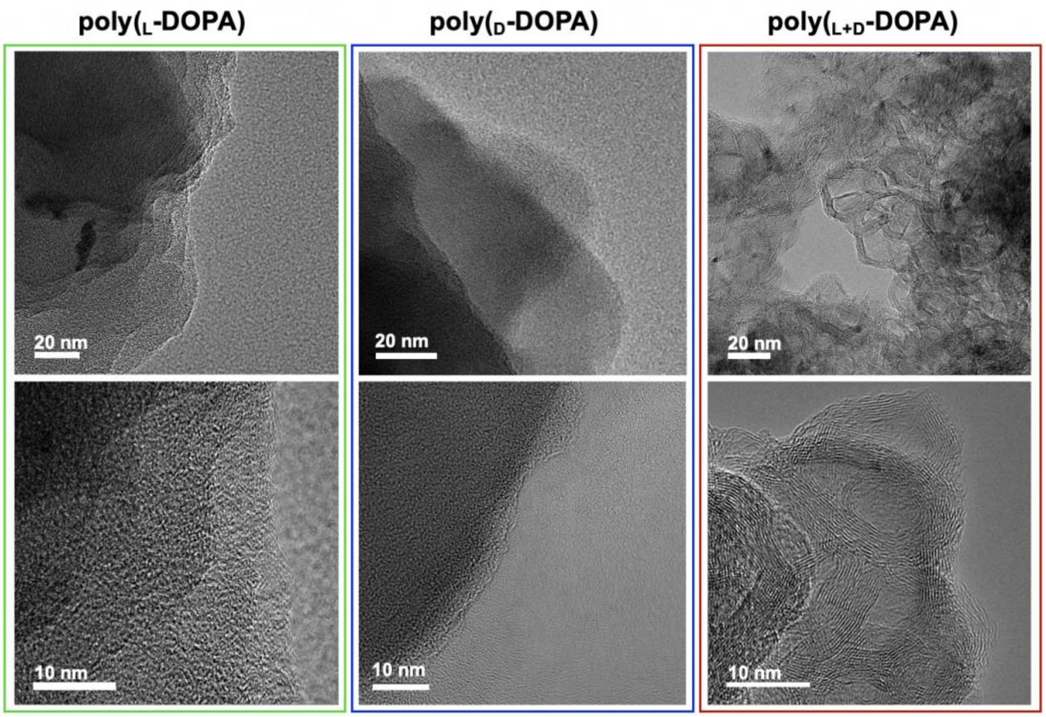

Supplementary Figure 11. Supplementary high-resolution transmission electron microscopy (TEM) images of the poly(L-DOPA), poly(D-DOPA), and poly(L+D-DOPA) solutions. It can be

clearly seen that all three molecules assemble into layer-by-layer structures, but only the poly(L+D-DOPA) molecule has the highly ordered fingerprint-like structure. The scale bars are 10 and 20 nm.

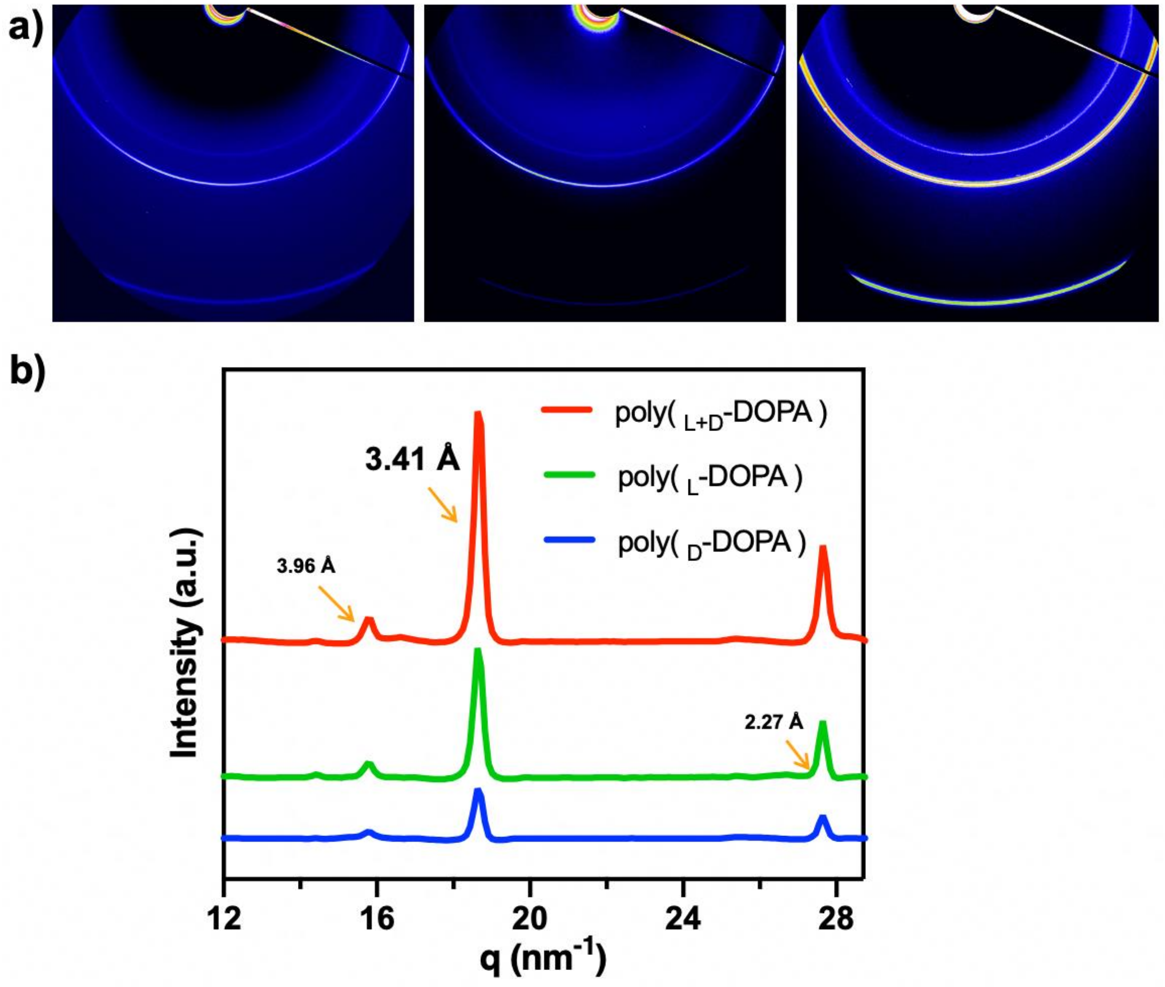

Supplementary Figure 12. The wide-angle X-ray scattering 2D patterns (a) and corresponding azimuthally integrated spectra (b) of poly(L-DOPA), poly(D-DOPA), and poly(L+D-DOPA) solutions. The oxidized DOPA solutions showed strong diffraction peaks corresponding to the spacing of 3.41 Å, similar to that of the melanin "local structure" produced by previous WAXS experiments, in which the 5,6-indolequinone units are arranged in planes by

π-stacking. [11] Among them, the heterochiral poly(L+D-DOPA) showed the strongest diffraction signal.

The WAXS of poly(DOPA) shows weaker diffraction peaks than XRD due to the low crystallinity in the solution, and the solids are more neatly arranged with the disappearance of the solvent.

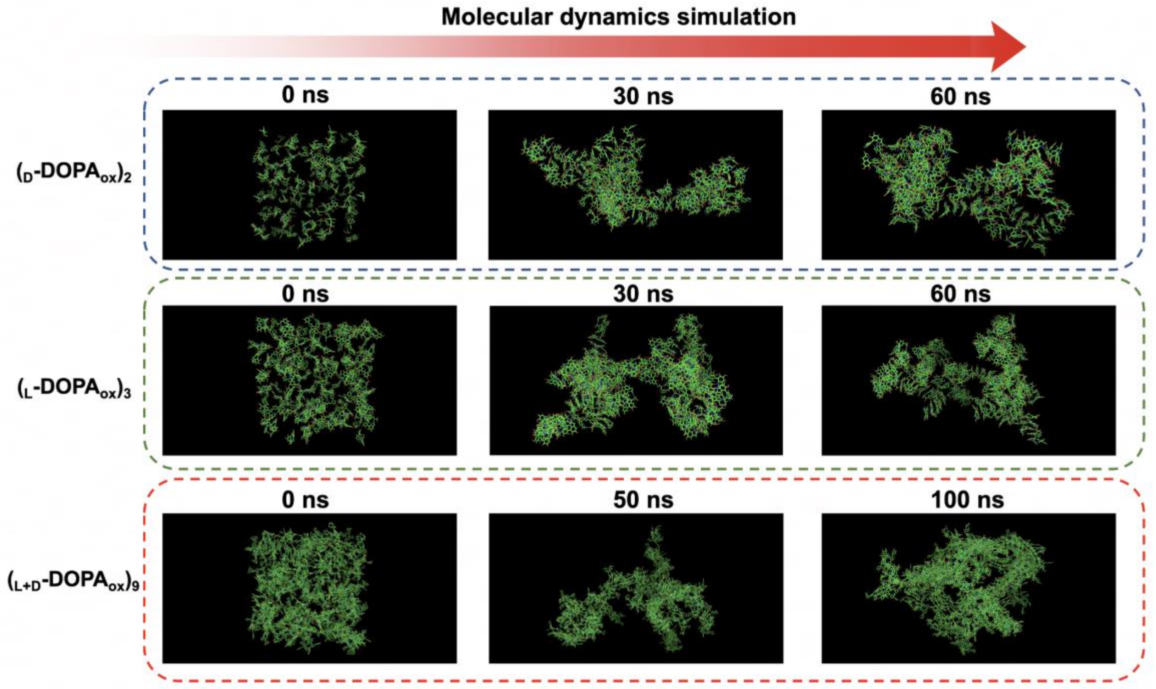

Supplementary Figure 13. Snapshots of the self-assembly process of 150 poly(DOPA) molecules in square box systems.

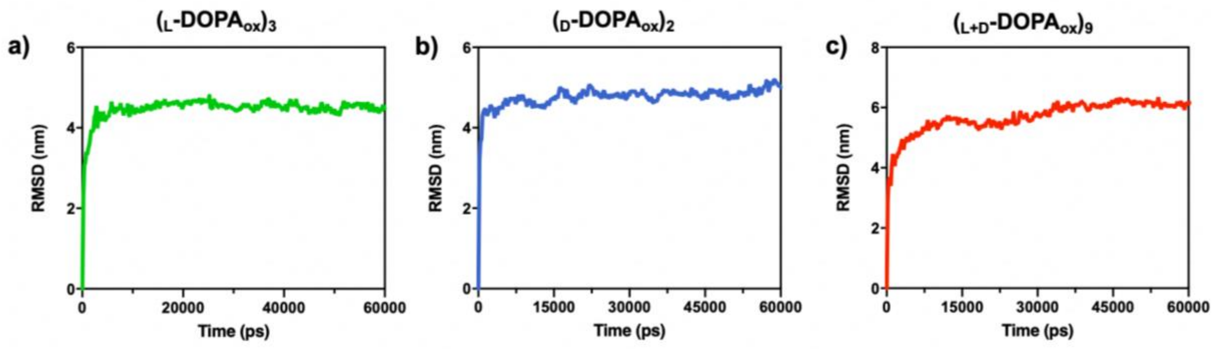

Supplementary Figure 14. The root mean square deviation (RMSD) curves of (L-DOPA$_{ox}$)$_3$ (a), (D-DOPA$_{ox}$)$_2$ (b) and (L+D-DOPA$_{ox}$)$_9$ (c). It proves the simulated systems have reached equilibrium at 60 ns and 100 ns, respectively.

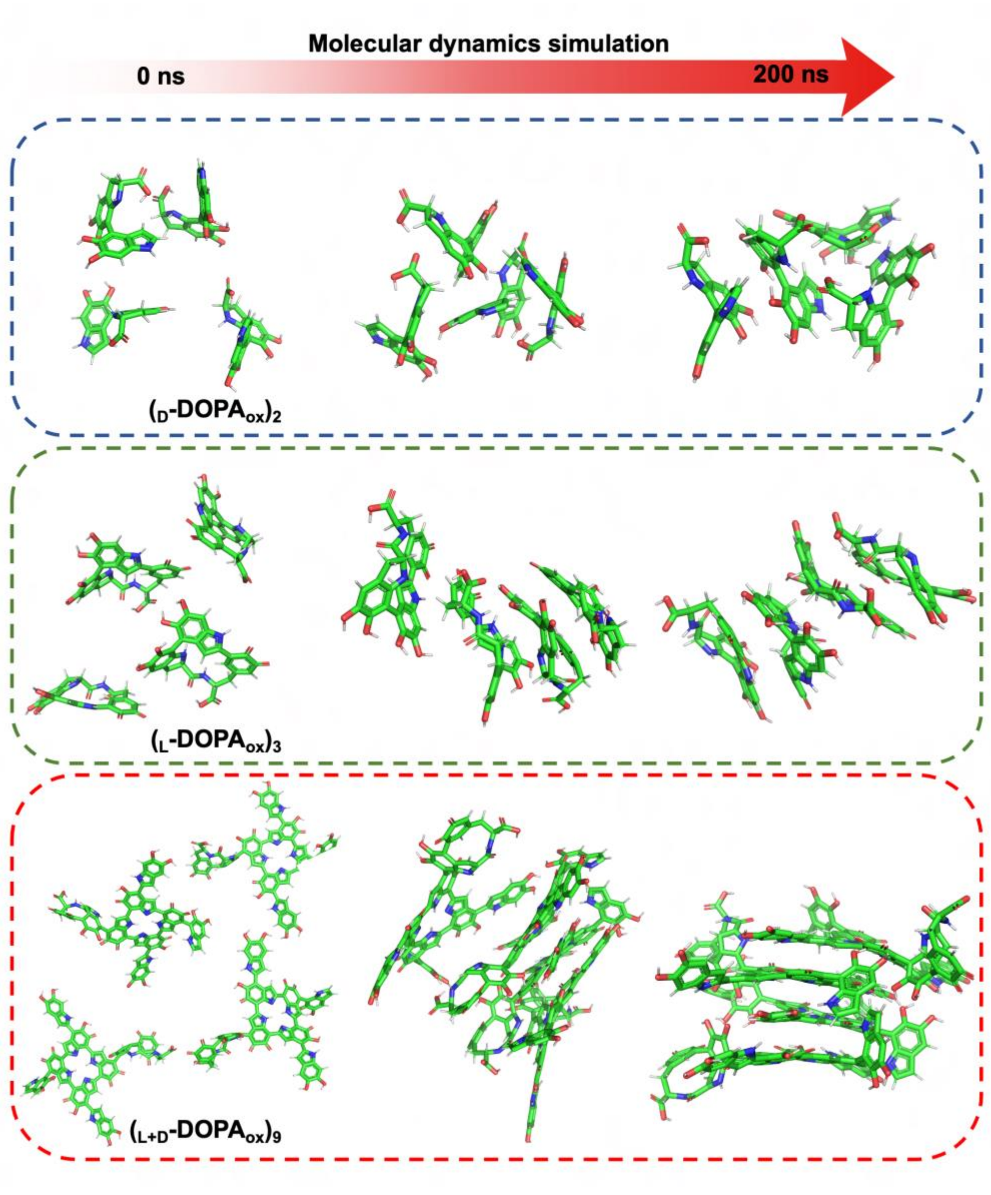

Supplementary Figure 15. Local snapshots of the self-assembly process of DOPA-oxidized polymers. The poly(DOPA) molecules are separated from each other in the initial configuration but quickly stack together to form a secondary structure.

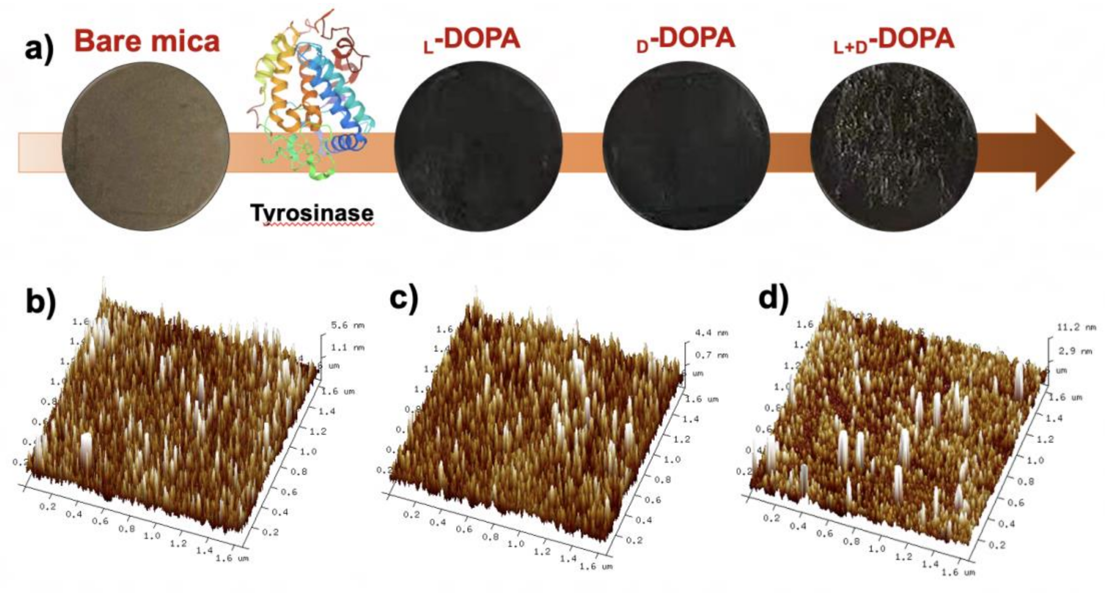

Supplementary Figure 16. Surface morphology analysis of DOPA coatings. a) Schematic illustration of the process of oxidative loading of chiral DOPA molecules on bare mica substrates. b-d) Three-dimensional topographic AFM images of the surface modified by (b) L-DOPA, (c) D-DOPA, and (d) L+D-DOPA. (Scanning area size = 1.6 × 1.6 μm).

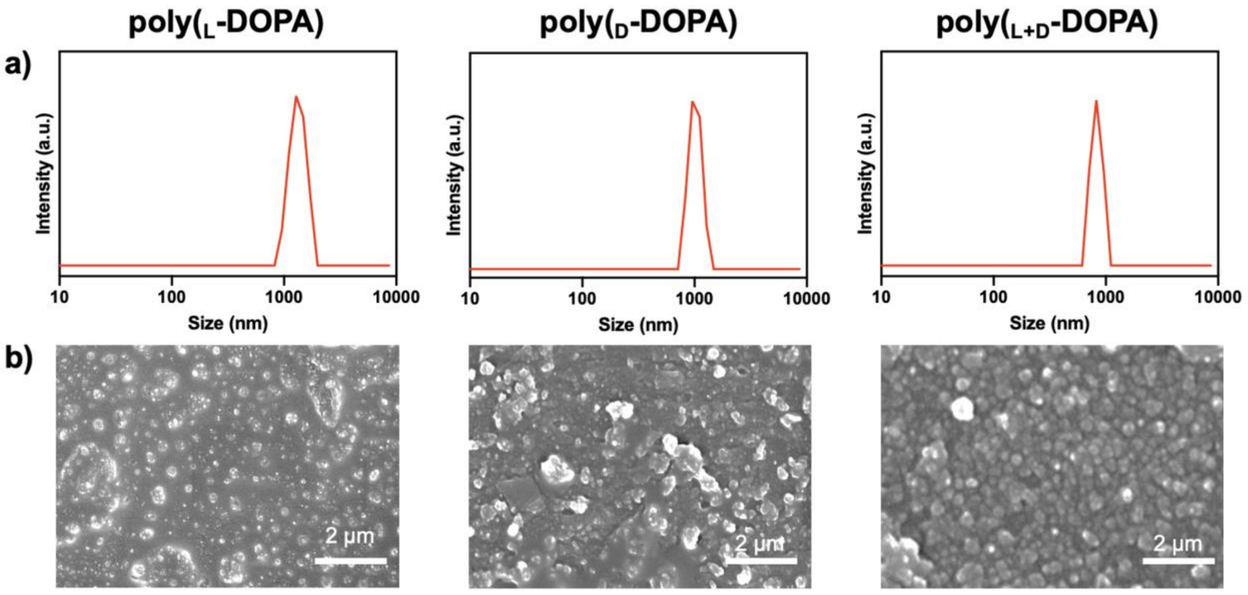

Supplementary Figure 17. (a) DLS and (b) SEM images of the nanoparticles assembled within the solutions after enzymatic oxidation. The scale bars are 2 μm.

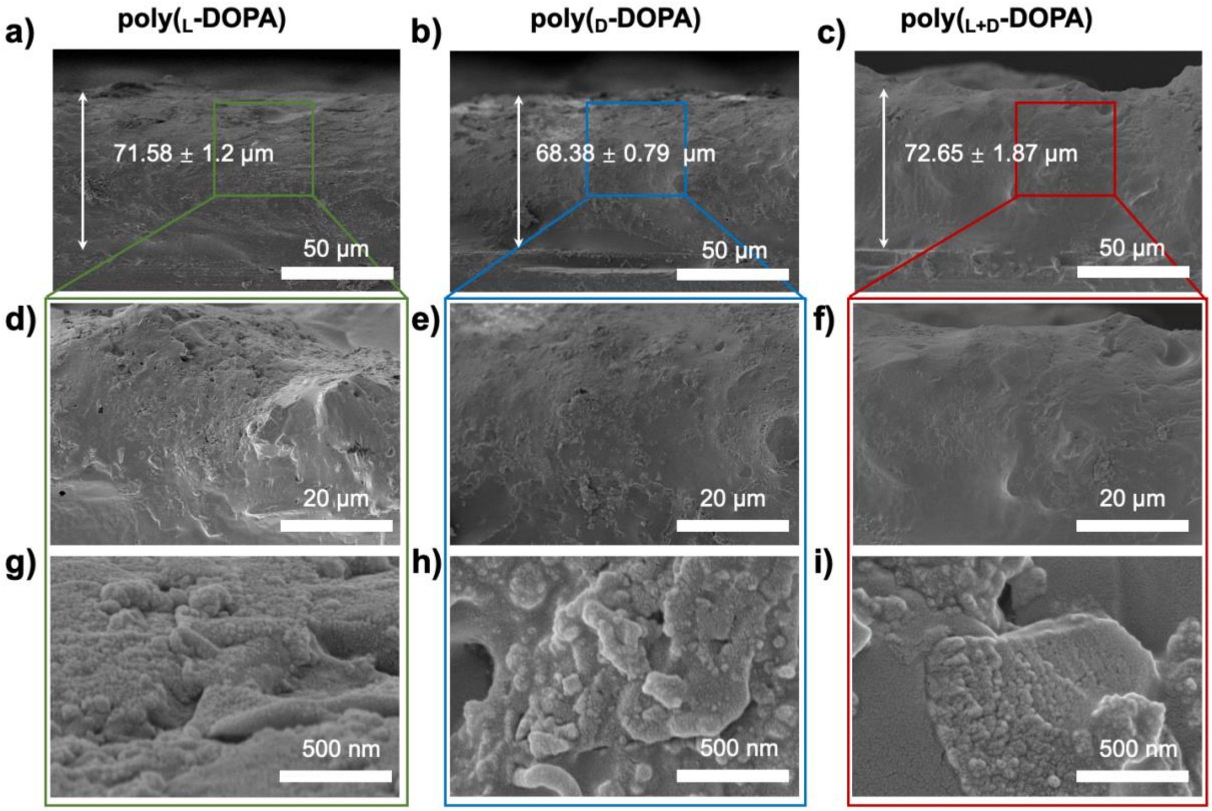

Supplementary Figure 18. Cross-section morphology and interior morphology of the three self-assembled poly(DOPA) films. a-c) The overall cross-section morphology of poly (DOPA) films when the scale bar is 50 μm, where poly (L+D-DOPA) (c) has the largest film thickness and poly (D-DOPA) (b) has the smallest thickness. d-i) The high-magnification SEM images of the film cross-section (The scale bar in d-f =20 μm, g-i = 500 nm) show that all three are formed by the stacking of polymer particles, but the poly (L+D-DOPA) film has fewer voids and more compact arrangement.

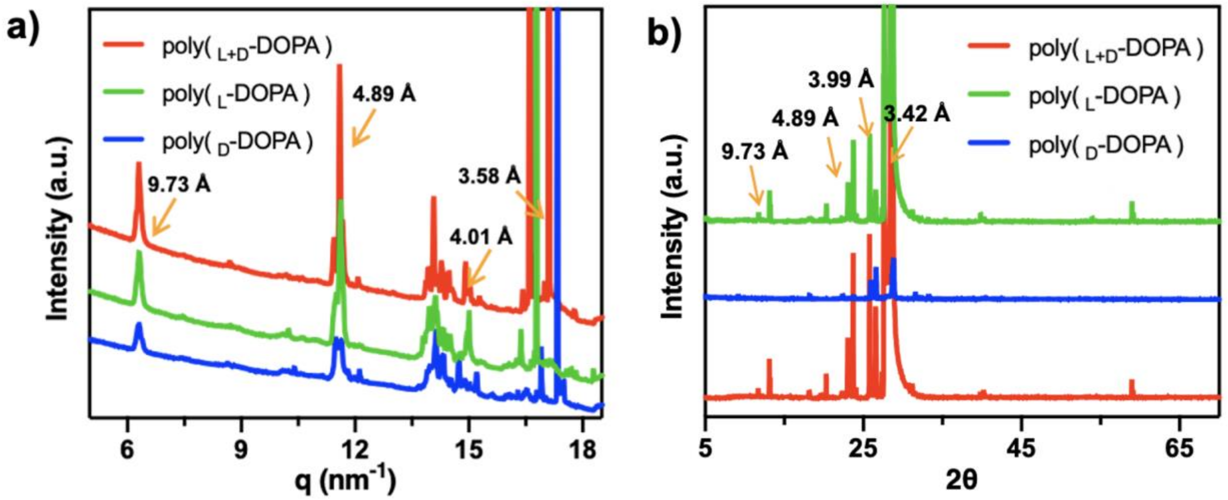

Supplementary Figure 19. Crystal structure analysis of poly(DOPA). a) 2D-GIWAXS data of enzymatic oxidative polymerization of DOPA in PBS (50 mM) and dried on a silicon substrate, corresponding azimuthally integrated spectra of poly(L-DOPA), poly(D-DOPA), and (d) poly(L+D-DOPA) shown in green, blue, and red, respectively. b) Powder X-ray diffraction XRD spectrum analysis of poly(DOPA). The X-ray diffraction of the dried assembled samples showed similar crystal structures and was consistent with the WAXS and 2D-GIWAXS data. All X-ray scattering results show that poly(L+D-DOPA) has a stronger alignment signal and diffraction peak, confirming that the alignment of the molecules in the mixture has changed.

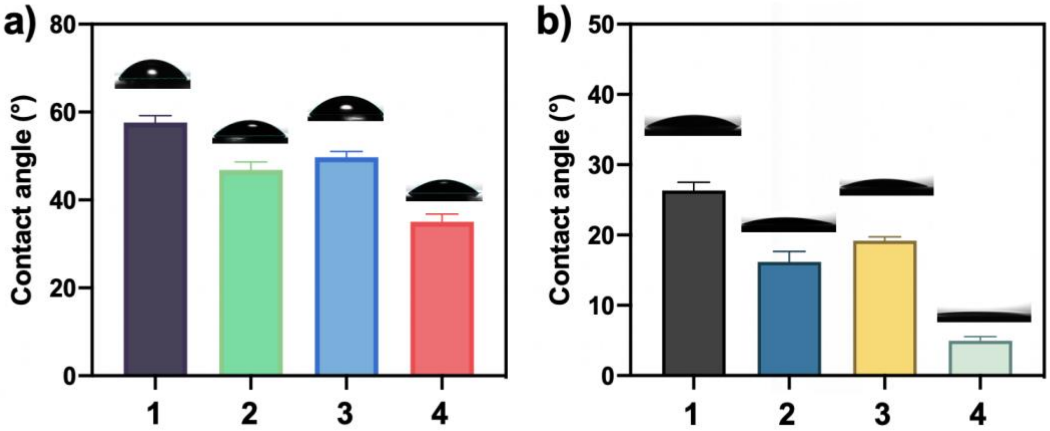

Supplementary Figure 20. Contact angles of different DOPA-coated surfaces. The substrate in the left picture is a glass slide (a), and the right is silica (b). water droplets on (1) bare substrate, (2) L-DOPA-coated, (3) D-DOPA-coated, and (4) L+D-DOPA hybrid coated at 25 ℃. Values were expressed as mean ± SD (n =3)

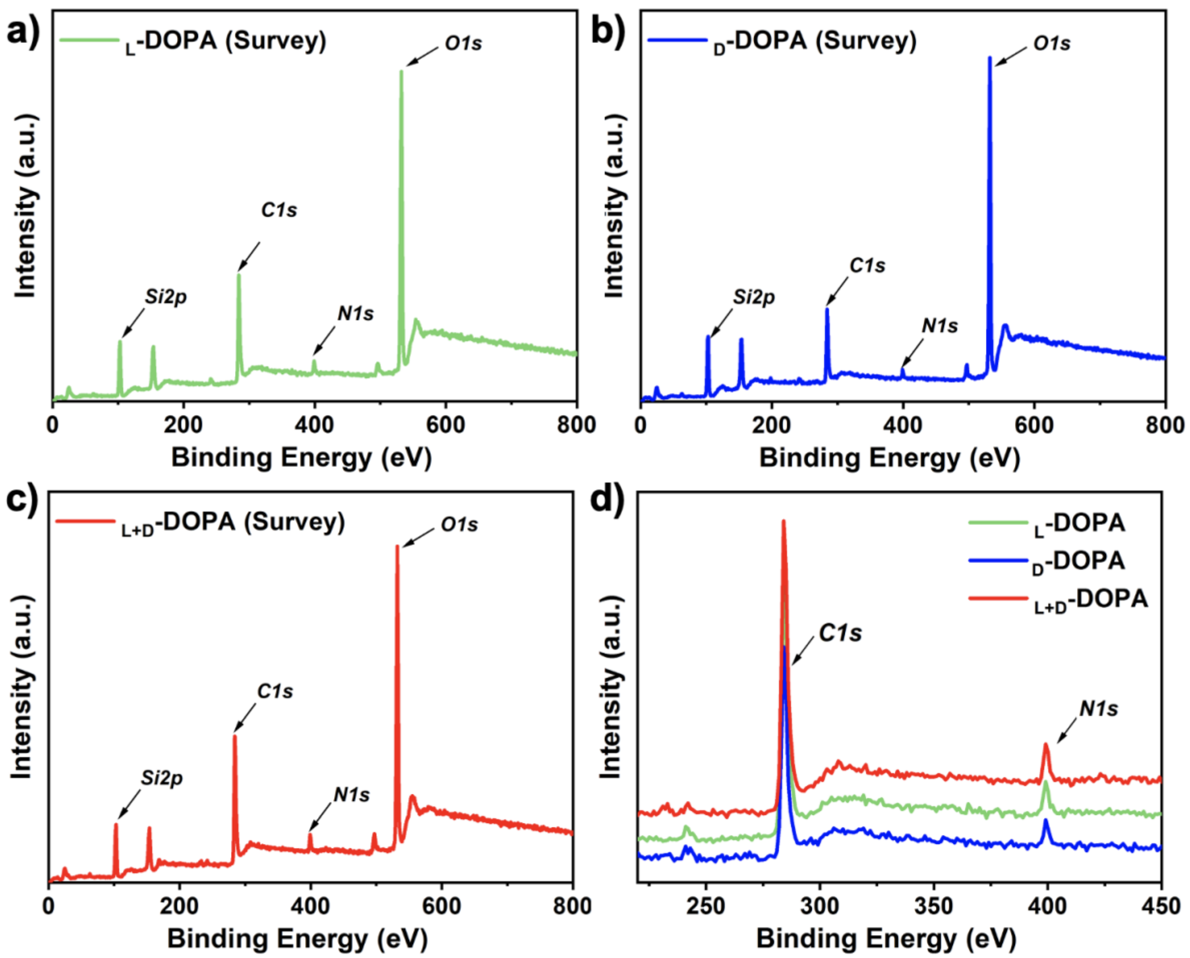

Supplementary Figure 21. a-c) Wide scan XPS spectrogram of the poly(L-DOPA) (a), poly(D-DOPA) (b), and poly(L+D-DOPA) (c) modifications on silica surfaces. d) Comparison of XPS element intensity of the three chiral DOPA modifications. C, O, N, and Si elements were detected by XPS. The high content of C suggested that polymers were successfully adsorbed on

the surface, as well as the existence of N. The high intensities of Si and O are attributed to the silica substrate.

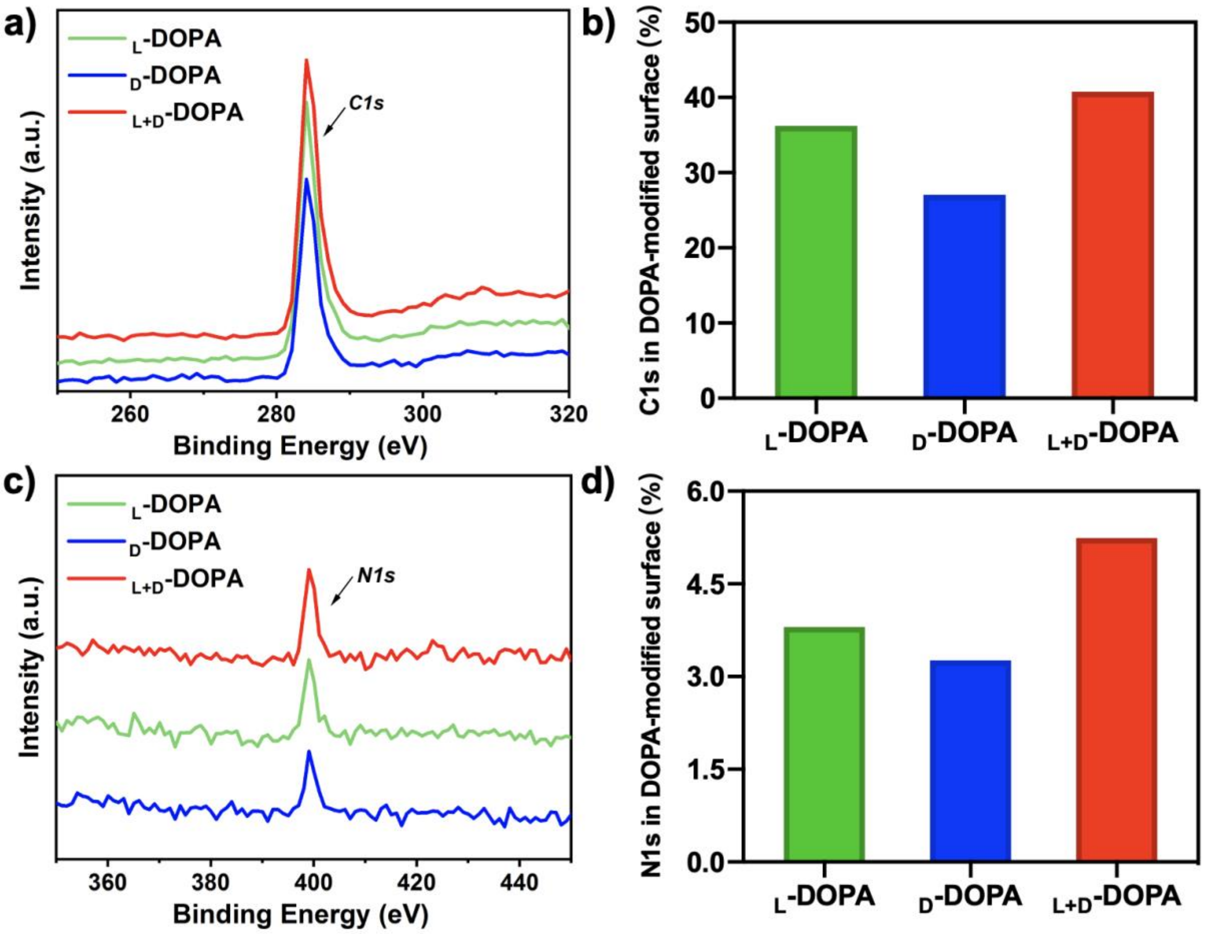

Supplementary Figure 22. a, c) XPS spectrogram monitoring the C/N element for chiral DOPA at the same adsorption concentrations for saturation adsorption. b, d) The amounts of C/N were collected and shown in the panel B for the C1S and D for the N1S. After the adsorption reached saturation, the C content in L-DOPA was 36.23%, and the N content was 3.8%, both of which were greater than the element content of the surface modified by D-DOPA with C of 27.05% and N of 3.26%. However, the chiral mixed L+D-DOPA obviously has a higher loading effect. The C element loading of 40.76% and the N element loading of 5.24% both confirmed its larger adsorption capacity, which is consistent with other experimental results.

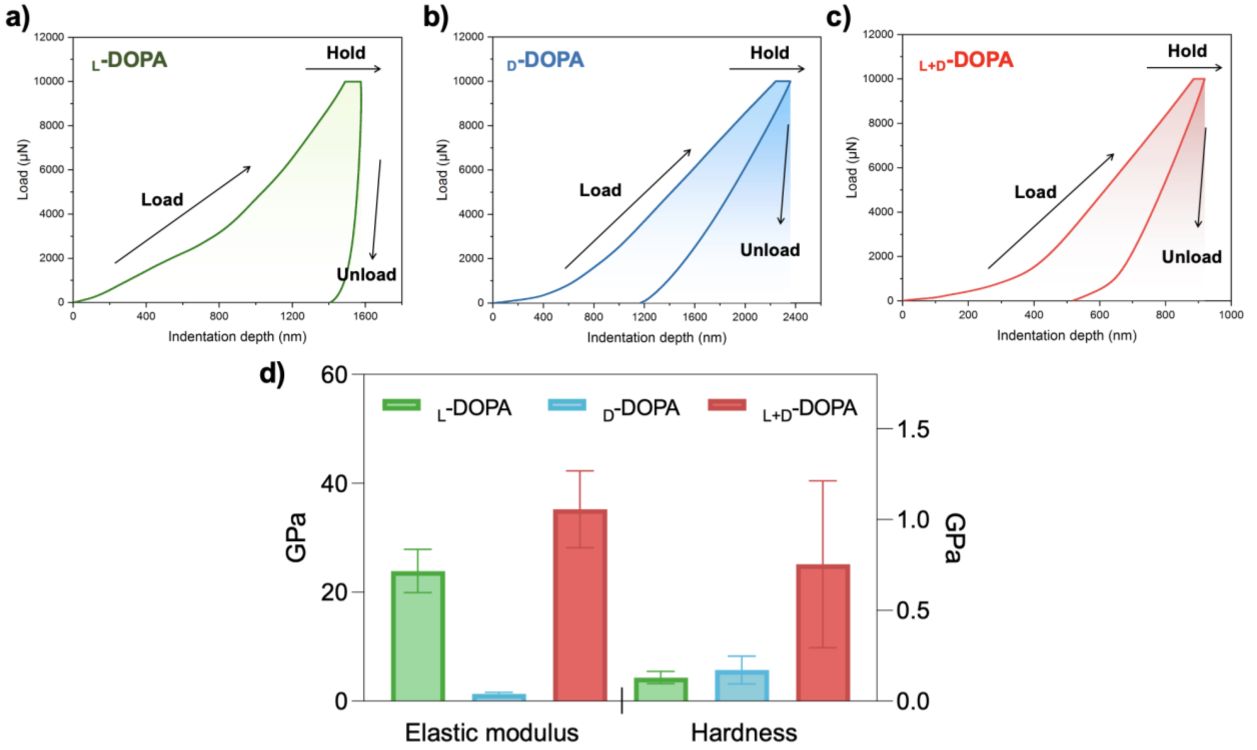

Supplementary Figure 23. Indentation curves obtained for the (a) poly(L-DOPA), (b) poly(L-DOPA), and (c) poly(L+D-DOPA) thin films and comparison graph for hardness and elastic modulus of three thin films (d). Data are presented as mean ± SD (n = 3 independent test results).

A previous study reported the mechanical response of polydopamine (PDA) films by nanoindentation experiments, with E = 2.3 ± 0.84 GPa for untreated films, which increased to >14 GPa upon calcination (600 °C).[12] And the highly oriented polydopamine film with a two-dimensional layered structure at the air/water interface showed excellent mechanical resilience with E = 13 ± 4 GPa and H = 0.21 ± 0.03 GPa due to its unique internal eumelanin-like supramolecular arrangement.[13] Possessing superior order and periodic organization like melanin, the poly(DOPA) film prepared here also exhibits excellent nanomechanical response. Since the limit of the maximum force applied by the nano indentation instrument used is limited to 10 mN, which is lower than the force applied by the AFM tip pressing downward, the elastic modulus

measured here differs from the Young's modulus value, but the racemic system also shows the highest mechanical strength.

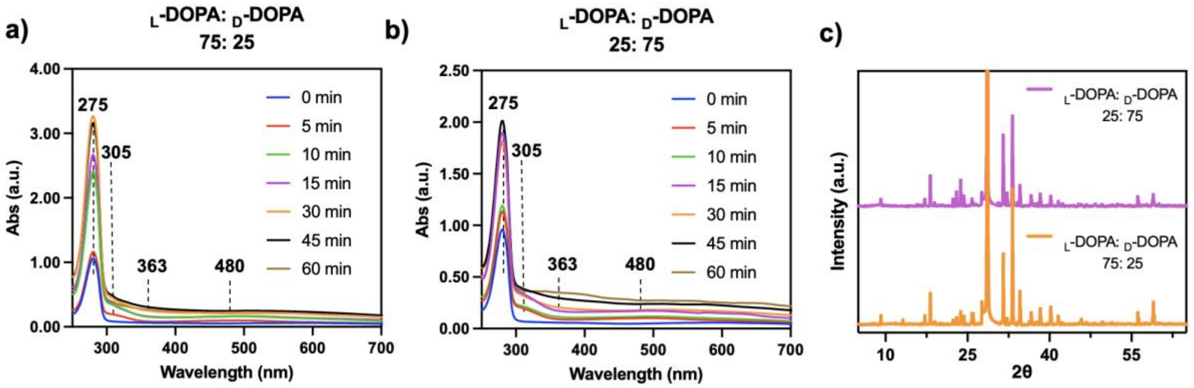

Supplementary Figure 24. UV–vis analysis and X-ray diffraction of chiral DOPA monomers with different doping ratios. a, b) In order to further investigate the effect of synergistic oxidation of chiral molecules on the properties of the assembled materials, solutions with L-DOPA and D-DOPA ratios of 25:75 (a) and 75:25 (b) (enantiomeric excess χ = ±50%; see supplementary methods), respectively, were also prepared. The UV absorption spectra showed that they also underwent oxidation to dopaquinone, which cyclization followed by redox exchange to form dopachrome and eventually to melanin-like pigments. c) Powder X-ray diffraction XRD spectroscopy of poly(DOPA)$_{25:75}$ and poly(DOPA)$_{75:25}$ showed the crystal structures similar to the racemic system, with the diffraction peak intensity similarly higher than that of the monochiral systems, confirming the changes in the arrangement of molecules in the mixture.

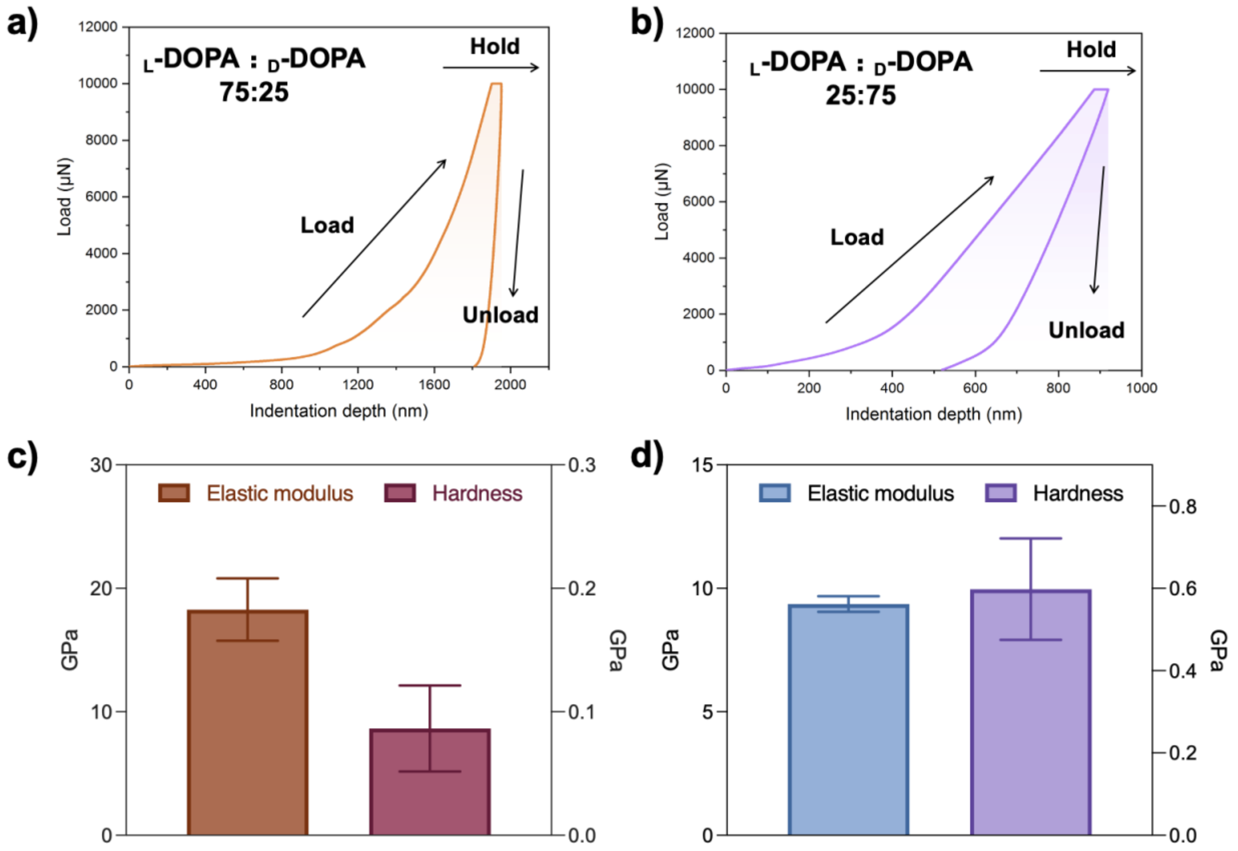

Supplementary Figure 25. Indentation curves and comparison graphs for hardness and elastic modulus obtained for the (a, c) poly(DOPA)$_{75:25}$ and (b, d) poly(DOPA)$_{25:75}$thin films. Data are presented as mean ± SD (n = 3 independent test results). Similar to other systems, both poly(DOPA)$_{25:75}$ and poly(DOPA)$_{75:\ 25}$ systems were equally uniformly loaded on the substrate and exhibited granular surface morphology. Nanoindentation experiments further confirmed that the poly(DOPA) films prepared here also exhibit superior nanomechanical response due to their superior melanin-like order and periodic organization. The indentation test results with the penetration depth kept under a load of 1000 μN showed general values of E=18.5 ± 3.6 GPa, H=0.09 ± 0.03 GPa and E=9.2 ± 0.3 GPa, H=0.59±0.14 GPa for poly(DOPA)$_{25:75}$ and poly(DOPA)$_{75:\ 25}$ samples, respectively. Compared with the homochiral D-DOPA system, the elastic properties and nanomechanical response for the coating films after oxidation of the mixed

chiral systems were improved, but they were both lower than the poly(L+D-DOPA) system. The above experiments confirmed that the racemic system leads to a more ordered sheet packing and tight arrangement of the films, which laid the foundation for its potential application in the coating field.

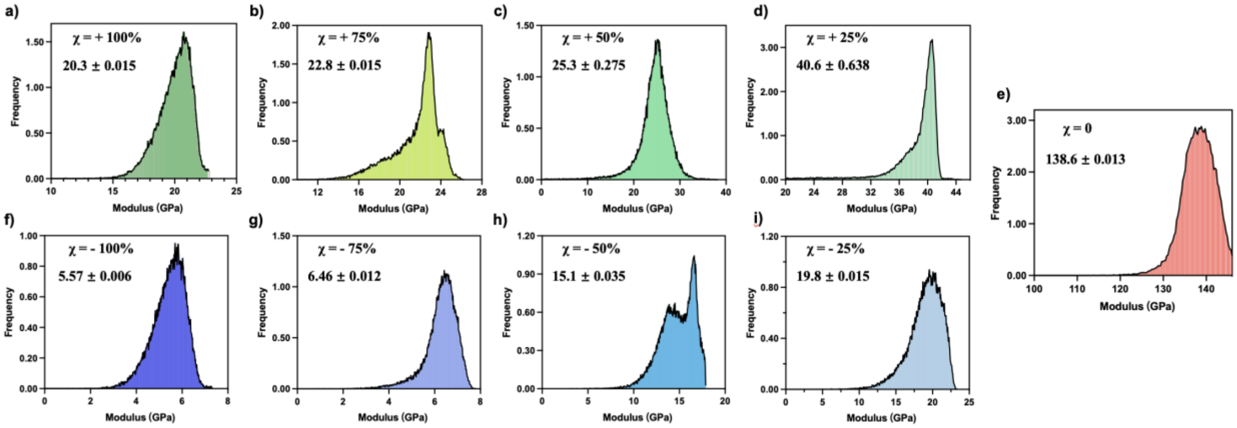

Supplementary Figure 26. a-f) AFM surface mechanical strength of poly(DOPA) films with different χ%, χ = +100%(a), +75%(b), +50%(c), +25%(d), 0%(e), -100%(f), -75%(g), -50%(h), -25%(i). The pure D-DOPA enantiomers (enantiomeric excess χ = -100%) with the lowest crystallinity and orderliness were measured with the lowest mechanical strength of 5.57 ± 0.006 GPa. With the increase of χ from -100 to 0, the mechanical properties of the coating films show an increasing trend and reach a maximum of 138.6 ± 0.013 GPa at the χ = 0, i.e. the racemic state. The mechanical strength of the system then falls back when the χ is further increased from 0 to +100, and the Young's modulus decreases from 40.6 ± 0.638 GPa at χ = +25% to 20.3 ± 0.015 GPa of the monochiral poly(L-DOPA) film.

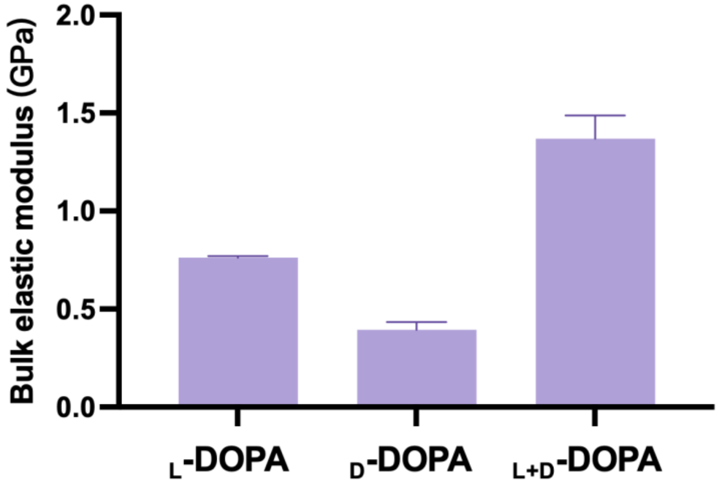

Supplementary Figure 27. Comparison of bulk elastic modulus between chiral poly(DOPA) films. Data are presented as mean ± SD (n = 3 independent test results).

Supplementary Tables

Supplementary Table 1. Comparison of kinetic parameters of different substrates catalyzed by tyrosinase.

Catalyst	Substrate	K_M (mM)	V_{max} (μM min^{-1})	V_{max}/K_M Ratio
Mushroom tyrosinase	L+D-DOPA	0.436	96.06	220.26
	L-DOPA	0.575	95.97	166.94
	D-DOPA	2.081	50.30	24.17

Supplementary references

1. Metz G, Wu XL, Smith SO. Ramped-Amplitude Cross Polarization in Magic-Angle-Spinning NMR. *J. Magn. Reson., Ser A* **110**, 219-227 (1994).
2. Fung BM, Khitrin AK, Ermolaev K. An improved broadband decoupling sequence for liquid crystals and solids. *J. Magn. Reson.* **142**, 97-101 (2000).
3. Riedel K, Herbst C, Leppert J, Ohlenschlager O, Gorlach M, Ramachandran R. Heteronuclear decoupling in rotating solids: Improving the efficacy of CNnv symmetry-based tanh/tan adiabatic RF pulse schemes. *Chem. Phys. Lett.* **429**, 590-594 (2006).
4. Tian S, Garcia-Rivera J, Yan B, Casadevall A, Stark RE. Unlocking the molecular structure of fungal melanin using 13C biosynthetic labeling and solid-state NMR. *Biochemistry* **42**, 8105-8109 (2003).
5. Grieco C, Kohl FR, Hanes AT, Kohler B. Probing the heterogeneous structure of eumelanin using ultrafast vibrational fingerprinting. *Nat. Commun.* **11**, 4569 (2020).
6. Heendeniya SN*, et al.* Therapeutic Efficacy of Nyctanthes arbor-tristis Flowers to Inhibit Proliferation of Acute and Chronic Primary Human Leukemia Cells, with Adipocyte Differentiation and in Silico Analysis of Interactions between Survivin Protein and Selected Secondary Metabolites. *Biomolecules* **10**, (2020).
7. Trott O, Olson AJ. Software News and Update AutoDock Vina: Improving the Speed and Accuracy of Docking with a New Scoring Function, Efficient Optimization, and Multithreading. *Journal of Computational Chemistry* **31**, 455-461 (2010).
8. Eberhardt J, Santos-Martins D, Tillack AF, Forli S. AutoDock Vina 1.2.0: New Docking Methods, Expanded Force Field, and Python Bindings. *J. Chem. Inf. Model.* **61**, 3891-3898 (2021).
9. Adasme MF*, et al.* PLIP 2021: expanding the scope of the protein-ligand interaction profiler to DNA and RNA. *Nucleic Acids Research* **49**, W530-W534 (2021).
10. Meng S, Kaxiras E. Theoretical models of eumelanin protomolecules and their optical properties. *Biophysical Journal* **94**, 2095-2105 (2008).
11. Cheng J, Moss SC, Eisner M. X-ray characterization of melanins--II. *Pigment Cell Res.* **7**, 263-273 (1994).

12. Li HQ, Xi JX, Zhao Y, Ren F. Mechanical properties of polydopamine (PDA) thin films. *Mrs Advances* **4**, 405-412 (2019).
13. Coy E, Iatsunskyi I, Colmenares JC, Kim Y, Mrowczynski R. Polydopamine Films with 2D-like Layered Structure and High Mechanical Resilience. *ACS Appl. Mater. Interfaces* **13**, 23113-23120 (2021).
